# Development and validation of an artificial intelligence-based model for diagnosing benign, borderline, and malignant adnexal masses

**DOI:** 10.1038/s41698-026-01320-5

**Published:** 2026-02-03

**Authors:** Yingnan Wu, Wenli Dai, Xiaoying Li, Shuang Zhang, Liping Gong, Jin Wang, Ailin Cui, Songxue Li, Manning Zhu, Shuang Dong, Yaoting Wang, Lei Zhou, Dexing Kong, Jing Zhao, Litao Sun

**Affiliations:** 1 Cancer Centre, Department of Ultrasound Medicine, Zhejiang Provincial People’s Hospital (Affiliated People’s Hospital), Hangzhou Medical College, Hangzhou, Zhejiang China; 2 Key Discipline of Zhejiang Province in Public Health and Preventive Medicine (First Class, Category A), Hangzhou Medical College, Hangzhou, Zhejiang China; 3 School of Mathematical Sciences, Zhejiang University, Zijingang Campus, Hangzhou, Zhejiang China; 4 Institute of Pathology and Southwest Cancer Center, Southwest Hospital, Third Military Medical University (Army Medical University) and Key Laboratory of Tumor Immunopathology, Ministry of Education of China, Chongqing, China; 5 Department of Ultrasound Medicine, Second Affiliated Hospital of Harbin Medical University, Harbin, Heilongjiang China; 6 Department of Ultrasound Medicine, Sichuan Provincial Maternity and Child Health Care Hospital, Chengdu, Sichuan China

**Keywords:** Oncology, Mathematics and computing

## Abstract

Classification of benign, borderline, and malignant adnexal masses is critical to effective clinical management, but remains a challenge. We developed Clinical-Ovarian Multi-Task Attention (Clinical-OMTA), an artificial intelligence model based on a dual-backbone architecture (benign vs. non-benign, and borderline vs. malignant) that integrates ultrasound, age, and Carbohydrate Antigen 125 (CA125) for multi-class classification. The model’s performance, generalisability, and clinical utility were evaluated. Retrospective data were collected from 23 hospitals (1882 patients for training, validation, and internal testing from 21 hospitals; 340 and 159 patients for external testing from two hospitals). In the external image dataset, Clinical-OMTA demonstrated comparable diagnostic performance to ADNEX (area under the receiver operating characteristic curve [AUC]: 0.950 vs. 0.953, 0.870 vs. 0.853, 0.930 vs. 0.938) and subjective assessment by an expert examiner (accuracy: 85.6% vs. 87.4%). While Clinical-OMTA supported multimodal integration, it did not outperform Ovarian Multi-Task Attention (OMTA) that trained only with images, indicating that including age and CA125 did not improve performance. Clinical-OMTA performed similarly across acquisition modes, equipment types, scanning methods, and different centres (accuracy: 79.9%–87.7%). With Clinical-OMTA as a decision support tool, radiologists showed significantly improved inter-reader agreement (kappa: 0.17–0.78 vs. 0.86–0.98) and diagnostic accuracy (72.3% vs. 88.0%). Clinical-OMTA appears generalisable and could be especially useful in low-resource or remote settings where expert ultrasound examiners are scarce.

## Introduction

Adnexal masses encompass benign, borderline, and malignant lesions with different clinicopathological characteristics, treatment protocols, and prognoses^1^. Malignant tumours are the most lethal gynecologic malignancies, necessitating referral to gynecologic oncology for thorough surgical staging and subsequent adjuvant chemotherapy^2^. Approximately 50% of borderline ovarian tumours occur in women of reproductive age. For young patients who wish to preserve their fertility, fertility-sparing surgery is the gold standard treatment approach, which does not compromise overall survival rates^3–5^. Patients with benign tumours may undergo conservative management or simple resection to avoid unnecessary costs and overtreatment^2^. Therefore, distinguishing benign from malignant lesions alone cannot enable appropriate treatment decisions, and diagnosis of adnexal masses with accurate multi-class classification is crucial for guiding individualised treatment decisions and improving quality of life.

Ultrasound, as a non-invasive imaging modality, is typically the preferred method for examining adnexal masses^6,7^. It can be used to discriminate benign, borderline, and malignant adnexal diseases and moreover to distinguish different histotypes of ovarian cancer with different treatments and prognoses. For example, Moro et al.^8^ proposed that a multilocular cyst with 2–10 locules suggested benign cystadenoma, while > 10 locules and papillary projections indicated a borderline tumour, and invasive mucinous gastrointestinal-type tumours typically appeared as multilocular-solid masses. In the same year, they proposed that papillary projections were the most typical ultrasound features of non-invasive (borderline and low grade) malignant serous tumours, whereas the presence of solid components but no papillations was the most representative image feature of invasive (low grade and high grade) serous tumours^9^. Ludovisi et al.^10^ described the serous surface papillary borderline ovarian tumours as a rare morphologic variant of serous ovarian tumours that were typically confined to the ovarian surface, as irregular solid lesions surrounding normal ovarian parenchyma. These findings reinforce the connection between intrinsic biological properties and ultrasound imaging characteristics.

However, the high complexity and diversity of ultrasound images present a significant challenge for diagnosing adnexal masses. This is particularly true for borderline tumours, as their ultrasound features often overlap with those of benign and invasive malignant tumours. Di Legge et al.^11^ illustrated the difficulty in distinguishing benign from very small borderline cysts with papillations. Flicek et al.^12^ demonstrated that borderline mucinous tumours may also present with pseudomyxoma peritonei, which can make it difficult to distinguish them from malignant mucinous carcinoma. Diagnosing these tumours via ultrasound is highly dependent on the experience and expertise of the operators^1^. Thus, subjective evaluation by experts has become the standard procedure for classifying adnexal masses. However, experts who can provide timely and accurate diagnoses and refer patients to appropriate clinical management are lacking^13^. Therefore, researchers have developed various clinical diagnostic models for adnexal masses based on clinical data, ultrasound features, and laboratory examination results, such as Malignant Risk Index (RMI)^14^, Gynecologic Imaging Reporting and Data System (GI-RADS)^15^, Simple Rules (SR)^16^, and Ovarian-adnexal Reporting and Data System (O-RADS)^17^, although limitations persist in assessing borderline tumours. The Assessment of Different NEoplasias in the adneXa (ADNEX) is the first model that can distinguish not only benign and malignant adnexal tumours, but also borderline ones, by incorporating clinical information such as age, Carbohydrate Antigen 125 (CA125), and type of centre, alongside ultrasound image features^1,18^. External validation studies have demonstrated its high diagnostic accuracy and widespread applicability in identifying adnexal tumours and assessing their risk^19,20^. However, the use of ADNEX still involves a subjective assessment of ultrasound-based features.

Recent scientific studies have demonstrated that machine learning (ML) can be applied to clinical data to solve the diagnostic problem of gynecologic oncology. Chiappa et al.^21^ conducted a pilot study in 2021, recruiting 241 women, and demonstrated that radiomics and machine learning allow us to identify patients with ovarian cancer. Subsequent studies by the same research group further supported the adoption of a radiomics- and ML-based approach for differential diagnosis of mesenchymal tumours^22^ and for predicting the response to neoadjuvant chemotherapy in advanced cervical cancer^23^. Recently, advancements in deep learning (DL) algorithms as a subset of ML have demonstrated significant benefits in ultrasound diagnosis and have become a research hotspot. DL can automatically learn subtle, complex, high-dimensional patterns from images and clinical data, eliminating the need for manual, operator-dependent feature engineering. In recent years, research on applying DL for diagnosing adnexal tumours has significantly progressed^24–35^. For example, Christiansen et al.^24^ showed that DL-based ultrasound analysis differentiates benign from malignant ovarian lesions with expert-level accuracy. Gao et al.^27^ developed and validated another ovarian cancer diagnosis model using data from 117,746 patients across ten Chinese hospitals and improved diagnostic accuracy for radiologists. Our team^32^ previously developed an automated DL framework, namely Ovarian Multi-Task Attention Network (OvaMTA), for ovary and ovarian mass detection, segmentation, and further diagnosis of ovarian masses based on ultrasound. Christiansen et al.^33^ developed and validated a transformer-based artificial intelligence (AI) model using 17,119 ovarian ultrasound images from 3652 patients across 20 international centres in eight countries, which demonstrated superior diagnostic accuracy to that of experts and non-experts and reduced expert referrals by 63%, thereby offering a solution for the global shortage of skilled ultrasound examiners.

However, there remains room for improvement. First, these studies primarily focused on distinguishing between benign and malignant tumours; however, research on the identification and diagnosis borderline adnexal tumours remains limited^25,28^. Borderline tumours are common in women of reproductive age and require accurate diagnosis to determine the optimal surgical approach for preserving fertility. Given the wide recurrence rate range of 5–46%, which reflects differences in surgical strategy and follow-up management, surveillance of borderline tumours remains essential^36–39^. Their ultrasound features overlap with those of both benign and malignant tumours, leading to potential misdiagnosis and delayed treatment^3^. Second, models trained on a single-centre dataset risk overfitting to centre-specific acquisition settings^40^. Multicentre data increase image diversity, thereby improving generalisability and robustness. Research on the generalisability and clinical utility of the AI models were limited. Third, existing studies that focused on the multi-class classification of benign, borderline, and malignant adnexal masses^25,28^ lacked evaluations of model generalisability across key clinical variables such as acquisition modes (static images vs. videos), equipment vendors, scanning methods (transvaginal vs. transabdominal), different centres, and histological subgroups.

In this multicentre retrospective cohort study, we trained and externally validated a model named Clinical-Ovarian Multi-Task Attention (Clinical-OMTA). This model was based on our previously published Multi-Task Attention Network (MTANet) architecture (extended with age and CA125 data) with two separate backbones (benign vs. non-benign; borderline vs. malignant) to automate the classification of benign, borderline, and malignant adnexal masses. We also compared the model’s diagnostic performance to that of ADNEX, the subjective assessment by an expert examiner, and the MTANet architecture training with images alone. Clinical-OMTA was developed to assist doctors of varying seniority in improving their diagnostic classification of adnexal masses, thereby better informing clinical decision-making. Thus, the performance and inter-reader agreement of AI-assisted junior, intermediate, and senior radiologists with and without AI-assistance were evaluated. We evaluated the model in different subgroups of the external test cohort, including acquisition modes (static images vs. videos), various equipment vendors, scanning methods (transvaginal vs. transabdominal), different centres, and histological subgroups, aiming to assess its clinical application and generalisation in clinical scenarios. We also sought to visually interpret the system’s “black box” and preliminarily explored whether its decision-making aligns with the subjective assessment by an expert examiner. The proposed model may assist in the classification of benign, borderline, and malignant adnexal masses and is a promising candidate for integration into clinical workflows, ultimately facilitating individualised treatment decisions.

## Results

### Patient characteristics

**Fig. 1:**
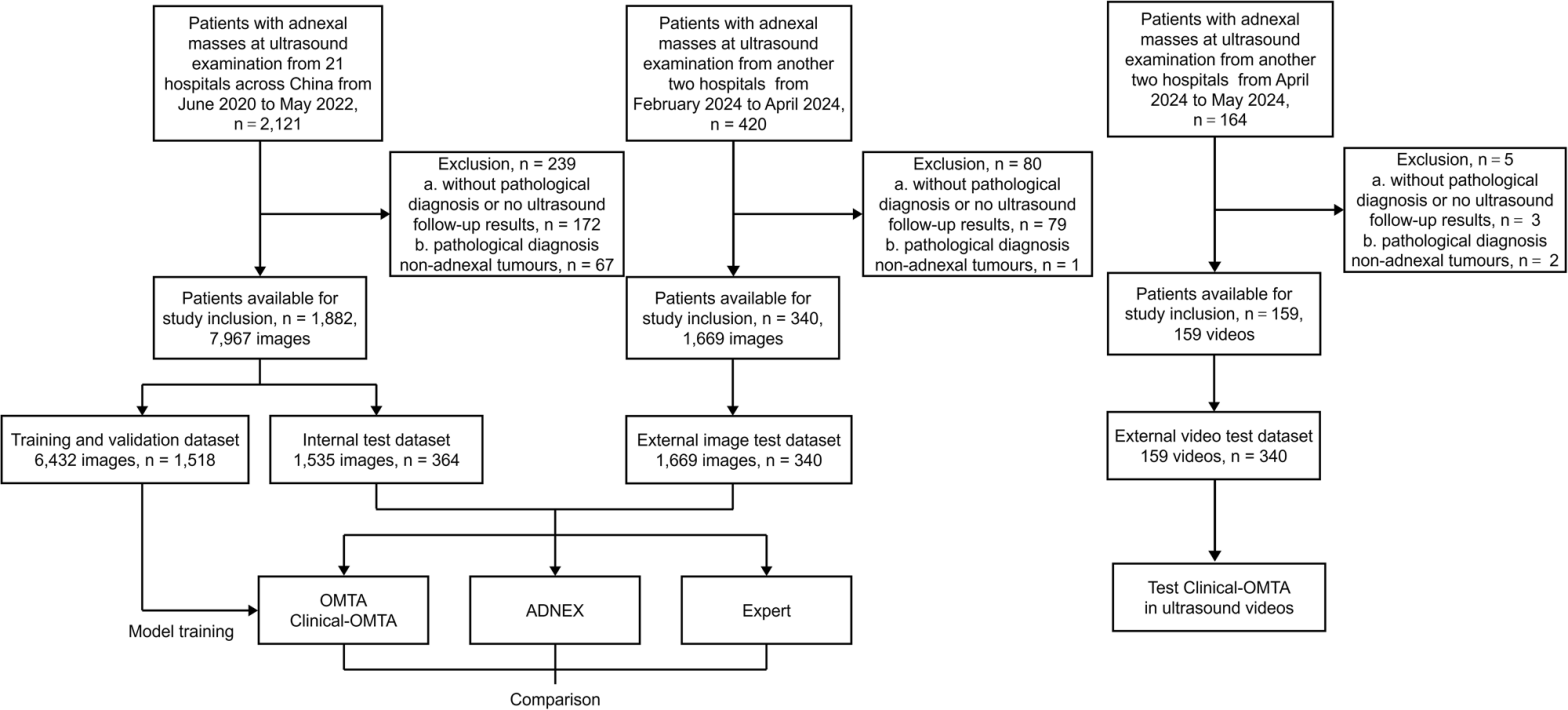
Flowchart showing the eligibility criteria and procedure of AI model evaluation. OMTA = Ovarian multi-task attention model, Clinical-OMTA = Clinical ovarian multi-task attention model, ADNEX = Assessment of Different NEoplasias in the adneXa.

A total of 2381 patients (median age 42 years [interquartile range (IQR): 31–53]) were included from 23 hospitals across 14 provinces, municipalities, and regions in China. This study included 9636 images (including 6432 images in the training and validation dataset, 1535 images in the internal test dataset, and 1669 images in the external test dataset) and 159 videos in the external test dataset. Patient characteristics across different datasets are listed in Table 1 and Fig. 1. These images and videos were acquired using 38 different commercial ultrasound systems from nine vendors. The top five vendors were GE Healthcare (1570 of 2381 patients, 65.9%), Samsung (385 of 2381 patients, 16.2%), Philips (291 of 2381 patients, 12.2%), Mindray (80 of 2381 patients, 3.4%), and Hitachi (33 of 2381 patients, 1.4%). The remaining four vendors together covered only 22 patients ( < 1.0%). Additionally, the five largest vendors accounted for > 97.0% of patients in the external image test dataset. In the external image test dataset, 17 device models were used, with seven models used in ≥ four patients and three in > 15 patients (Table S1). Nearly 69.3% (1649/2381) of patients had evaluated CA125 levels.

**Table 1:** Baseline characteristics of patients

Characteristic	Training and validation dataset	Internal test dataset	External test dataset (image)	External test dataset (video)
No. of patients	1518	364	340	159
No. of images/videos	6432	1535	1669	159
Age (years)	41.55 ± 13.78 (12–86)	43.24 ± 14.78 (15–81)	41.20 ± 14.70 (11–75)	43.40 ± 14.65 (17–81)
CA125 (U/mL)	304.88 ± 1,225.26 (4.6–25,000.0)	220.88 ± 551.57 (2.0–4707.0)	212.78 ± 555.20 (1.8–9713.8)	253.22 ± 634.76 (0–4000.0)
Tumour type				
Benign	961 (63.3)	183 (50.3)	226 (66.5)	93 (58.5)
Borderline	135 (8.9)	52 (14.3)	37 (10.9)	18 (11.3)
Malignant	422 (28.8)	129 (35.4)	77 (22.6)	48 (30.2)
Histology				
Germ cell	293 (19.3)	63 (17.3)	146 (42.9)	15 (9.4)
Serous	411 (27.0)	129 (35.4)	68 (20.0)	58 (36.5)
Mucinous	104 (6.9)	45 (12.3)	36 (10.6)	31 (19.5)
Sex cord stromal tumour	61 (4.0)	24 (6.6)	20 (5.8)	11 (6.9)
Mixed^a^	73 (4.8)	12 (3.3)	20 (5.8)	6 (3.8)
Clear cell	29 (1.9)	10 (2.8)	4 (1.2)	4 (2.5)
Endometrioid	35 (2.3)	10 (2.8)	6 (1.8)	6 (3.8)
Metastatic adnexal tumour	15 (1.0)	9 (2.5)	4 (1.2)	7 (4.4)
Others^b^	532 (35.0)	62 (17.0)	36 (10.6)	21 (17.6)

Continuous data are presented as mean ± standard deviation (SD) with range in parentheses. Categorical variables are shown as numbers; data in parentheses are percentages. The age information of all the patients was known. In the training, validation, and internal test datasets, 1249 (66.4%) patients had the CA125 test. In the external image test dataset, 241 (70.9%) patients had the CA125 test. In the external video test dataset, all patients underwent CA125 testing. Six patients had two tumours with the same pathology, including four patients in training dataset, one patient in the internal test dataset, and one in the external image test dataset.

*CA125* Carbohydrate antigen 125.

^a^Mixed histology of adnexal mass indicates that the mass simultaneously contains multiple pathological subtypes.

^b^Other histology encompassed mesenchymal tumour and Brenner tumour, and others.

### Comparison of diagnostic performance among OMTA, Clinical-OMTA, ADNEX, and subjective assessment by an expert examiner

**Fig. 2:**
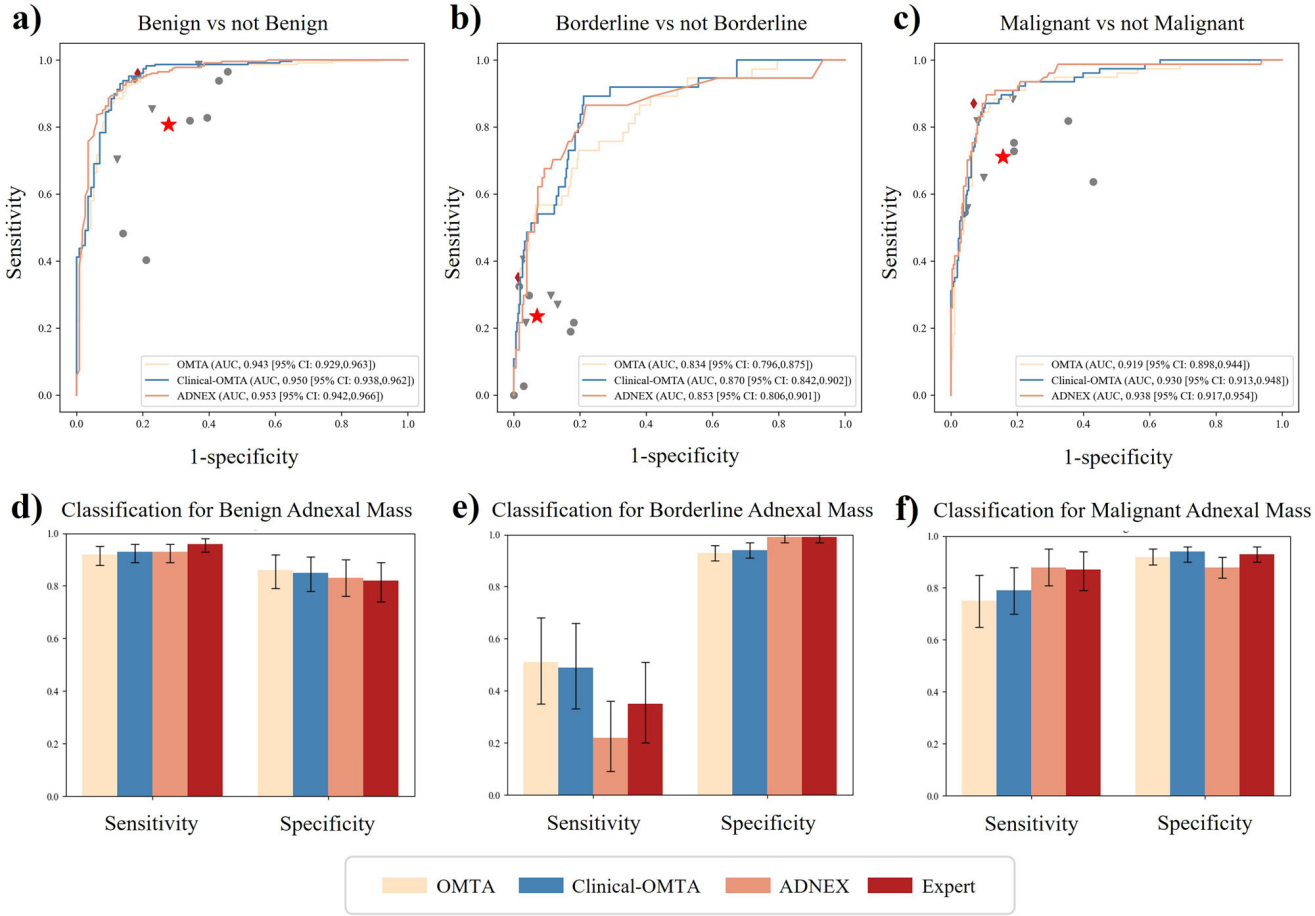
Diagnostic performance of OMTA, Clinical-OMTA, ADNEX, subjective assessment by an expert examiner, and radiologists with varying levels of experience in distinguishing benign, borderline, and malignant adnexal tumours in the external test dataset. **a**–**c** show ROC curves of OMTA, Clinical-OMTA, and ADNEX. The red star represents the average performance of the 11 radiologists, where sensitivities and specificities were averaged across radiologists. Grey circles represent the performance of junior radiologists, grey triangles represent intermediate radiologists, and red diamonds represent subjective assessment by an expert examiner. ROC curves were based on 340 patient-level predictions in the external test datasets. **d**–**f** show bar charts of sensitivity and specificity; 95% CIs were obtained by patient-level bootstrap (5000 resamples). OMTA = Ovarian multi-task attention model, Clinical-OMTA = Clinical ovarian multi-task attention model, ADNEX = Assessment of Different NEoplasias in the adneXa, AUC = Area under the receiver operating characteristic curve, ROC = Receiver operating characteristic.

The diagnostic performance of Ovarian Multi-Task Attention (OMTA), Clinical-OMTA, ADNEX, and subjective assessment by an expert examiner are presented in Fig. 2, S1, S2 and Tables S2, S3. In the internal test dataset, areas under the receiver operating characteristic curves (AUCs) for diagnosing benign, borderline, and malignant tumours using Clinical-OMTA were 0.938 (95% CI: 0.924, 0.955), 0.839 (95% CI: 0.808, 0.884), and 0.941 (95% CI: 0.929, 0.955), respectively. In the external image test dataset, the AUCs for diagnosing benign, borderline, and malignant tumours using Clinical-OMTA were 0.950 (95% CI: 0.938, 0.962), 0.870 (95% CI: 0.842, 0.902), and 0.930 (95% CI: 0.913, 0.948), respectively.

In our study, AUCs of Clinical-OMTA for classifying benign, borderline, and malignant adnexal masses were overall comparable to those of ADNEX, in both the internal test dataset (*P* = 0.736; *P* = 0.397; *P* = 0.027) and the external image test dataset (*P* = 0.827; *P* = 0.700; *P* = 0.621). The accuracy of Clinical-OMTA in distinguishing benign, borderline, and malignant adnexal tumours was comparable to that of ADNEX (79.4% [95% CI: 75.3, 83.5] vs. 79.4% [95% CI: 75.0, 83.5], *P* = 1.000; 85.6% [95% CI: 81.8, 89.1] vs. 84.1% [95% CI: 80.0, 87.9], *P* = 0.583) and similar to those of expert subjective assessment (79.4% [95% CI: 75.3, 83.5] vs. 81.6% [95% CI: 77.5, 85.4], *P* = 0.389; 85.6% [95% CI: 81.8, 89.1] vs. 87.4% [95% CI: 83.5, 90.6], *P* = 0.441) in both the internal and external image test datasets. The binary net re-classification improvement (NRI)^41^ was used to assess whether adding age and CA125 improved performance over the image-only OMTA model. Across all three binary tasks (benign vs. non-benign, borderline vs. non-borderline, and malignant vs. non-malignant), Clinical-OMTA showed no significant re-classification improvement in either the internal (NRI = -1.12%, 5.13%, -0.12%; *P* = 0.546, 0.116, 0.904) or external image test datasets (0.02%, 2.37%, 1.59%; *P* = 1.000, 0.584, 0.584).

The calibration curves of Clinical-OMTA showed good correspondence between the predicted risk and the actual observed proportion of benign, borderline and malignant adnexal masses in both the internal and external image test datasets, indicating well-calibrated predictions (Fig. S3a–f). This indicated that the model’s confidence was correlated with the likelihood of it making a correct prediction.

### The performance and inter-reader agreement of Clinical-OMTA in assisting radiologists of differing seniority

**Fig. 3:**
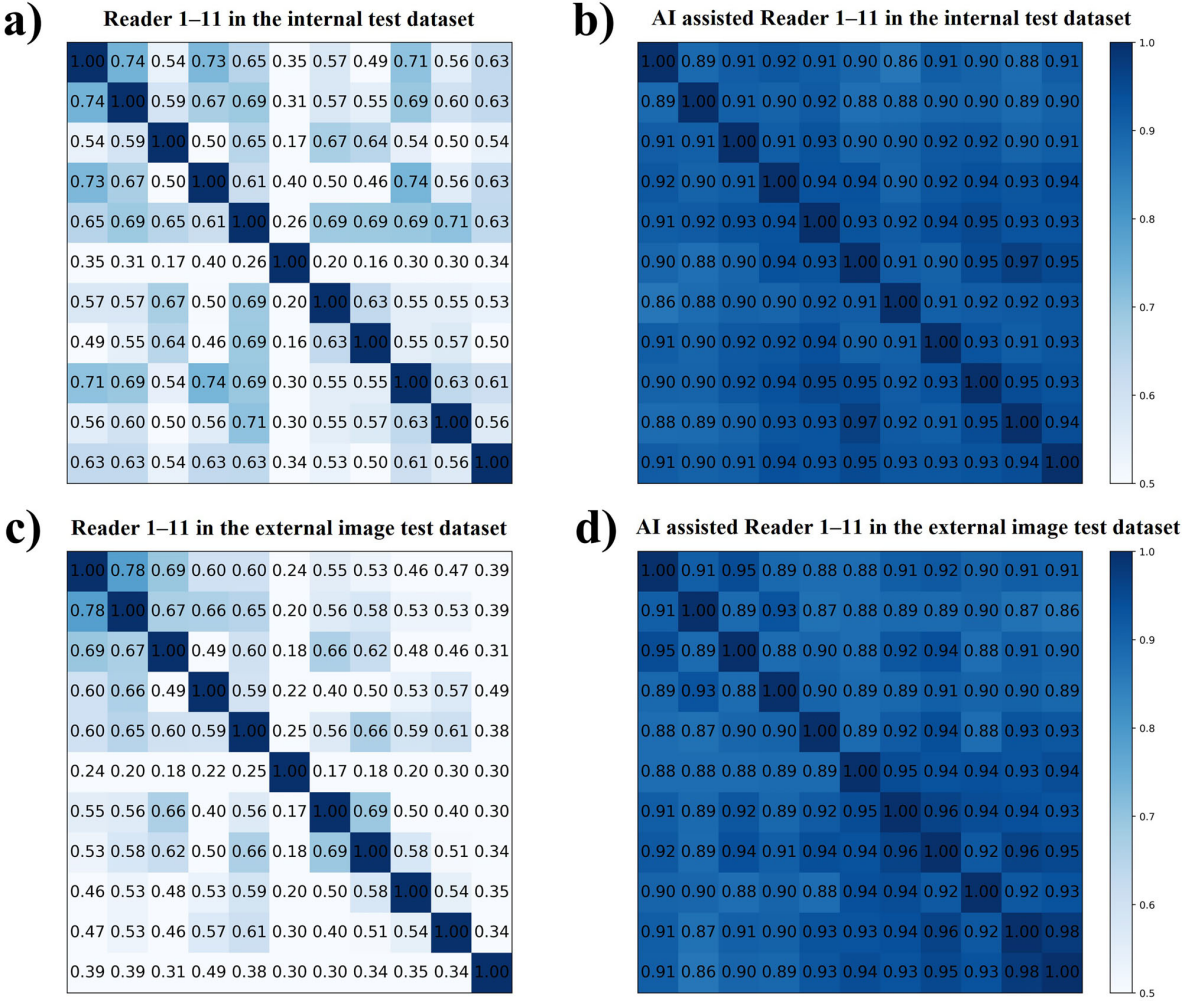
Clinical-OMTA model improved radiologists’ inter-reader agreement. **a** shows the matrix of kappa values of 11 radiologists’ diagnoses in the internal test dataset. **b** shows the matrix of kappa values of the diagnoses by 11 radiologists assisted by the Clinical-OMTA model in the internal test dataset. **c** shows the matrix of kappa values of 11 radiologists’ diagnoses in the external image test dataset. **d** shows the matrix of kappa values of the diagnoses by 11 radiologists assisted by the Clinical-OMTA model in the external image test dataset. Each value in the matrices represents the inter-reader agreement between two radiologists. On the axes of kappa matrices, radiologists 1–11 were arranged in the order from top to bottom and from left to right. OMTA = Ovarian multi-task attention model, Clinical-OMTA = Clinical ovarian multi-task attention model.

The overall diagnostic accuracy of Clinical-OMTA-assisted radiologists (*n* = 11) in the internal and external image test datasets revealed significant gains of 14.7 and 15.7 percentage points, respectively (69.8% [95% CI: 68.4, 71.3] vs. 84.5% [95% CI: 83.4, 85.6], *P* < 0.001; 72.3% [95% CI: 70.9, 73.7] vs. 88.0% [95% CI: 86.9, 89.0], *P* < 0.001) (Table S4). Among these radiologists, in the internal and external image test datasets, the diagnostic accuracy of junior radiologists demonstrated significant gains of 17.7 and 20.1 percentage points (65.2% [95% CI: 63.2, 67.2] vs. 82.9% [95% CI: 81.3, 84.5], *P* < 0.001; 66.3% [95% CI: 64.3, 68.2] vs. 86.4% [95% CI: 85.0, 87.9], *P* < 0.001), while those of intermediate radiologists showed significant gains of 12.5 and 11.8 percentage points, respectively (73.8% [95% CI: 71.5, 76.1] vs. 86.3% [95% CI: 84.6, 88.1], *P* < 0.001; 77.6% [95% CI: 75.4, 79.8] vs. 89.4% [95% CI: 87.8, 91.0], *P* < 0.001) and those of the expert radiologist showed gains of 4.9 and 2.3 percentage points, respectively (81.6% [95% CI: 77.5, 85.4] vs. 86.5% [95% CI: 82.7, 89.8], *P* = 0.004; 87.4% [95% CI: 83.5, 90.6] vs. 89.7% [95% CI: 86.2, 92.6], *P* = 0.014). Tables S5–S8 present the detailed performance of the 11 radiologists, with and without the assistance of Clinical-OMTA in the internal and external image test datasets. Reader-level change analysis revealed that Clinical-OMTA assistance predominantly corrected errors, but also introduced new errors in the internal and external datasets (Tables S9 and S10). Without the assistance of Clinical-OMTA, inter-reader kappa values were 0.17–0.74 and 0.17–0.78 in the internal and external image test datasets, respectively, indicating fair to moderate agreement. With the assistance of Clinical-OMTA, inter-reader agreement improved, as evidenced by kappa values of 0.86–0.97 and 0.86–0.98 in the internal and external image test datasets, respectively, indicating good agreement (Fig. 3).

### Generalising Clinical-OMTA to different scenarios

In the external video test dataset, we conducted comparable experiments on three approaches, including a fully automated video pipeline, expert manual correction, and three representative frames (Table S11). For external videos with expert manual correction, AUCs of Clinical-OMTA for diagnosing benign, borderline, and malignant tumours were 0.907 (95% CI: 0.888, 0.927), 0.903 (95% CI: 0.879, 0.926), and 0.952 (95% CI: 0.939, 0.968) (Figs. S4, S5) with an accuracy of 79.9% (95% CI: 73.6, 86.2). The calibration curve of Clinical-OMTA also showed good correspondence between the predicted risks of benign, borderline, and malignant adnexal masses and their actual observed proportions in the external video test dataset, indicating well-calibrated predictions. The calibration curve for borderline cases exhibited wider confidence intervals, likely due to the relatively limited number of borderline cases in the dataset (Fig. S3g–i).

Thus, Clinical-OMTA maintains high diagnostic performance at the video level. To assess the generalisability of Clinical-OMTA, we evaluated its diagnostic performance across external test datasets comprising images from two different centres, multiple ultrasound vendors, and two scanning methods. The model has achieved similar accuracy across acquisition modes (79.9%–85.6%), equipment types (85.1%–87.7%), scanning methods (84.6%–85.9%), and different centres (85.4%–85.6%) (Tables 2 and S12). Subgroup analysis by histologic subtype (Table S13) revealed that Clinical-OMTA demonstrated good diagnostic performance for germ cell tumours, serous tumours, and other tumours (accuracy: 94.5% [95% CI: 90.8, 98.2], 83.8% [95% CI: 75.1, 92.6], and 82.0% [95% CI: 71.4, 92.7]). In contrast, accuracy was lower for mucinous tumours (69.4% [95% CI: 54.4, 84.5]), sex cord stromal tumours (75.0% [95% CI: 56.0, 94.0]), and mixed tumours (75.0% [95% CI: 56.0, 94.0]).

**Table 2:** Generalisation of clinical-OMTA based on acquisition modes, vendors, scanning methods, and different centres in external image and video test datasets

	Accuracy (%)	Benign AUC	Borderline AUC	Malignant AUC
Acquisition modes				
Static images	85.6 (291/340) [81.8, 89.1]	0.950 [0.938, 0.962]	0.870 [0.842, 0.902]	0.930 [0.913, 0.948]
Videos^a^	79.9 (127/159) [73.4, 86.2]	0.907 [0.891, 0.930]	0.903 [0.882, 0.922]	0.952 [0.941, 0.964]
Vendors				
GE	85.1 (182/214) [80.3, 89.8]	0.939 [0.901, 0.973]	0.859 [0.775, 0.926]	0.924 [0.876, 0.964]
Philips	87.2 (34/39) [76.7, 97.7]	0.954 [0.865, 1.000]	0.841 [0.537, 1.000]	0.994 [0.970, 1.000]
Mindray	87.7 (50/57) [79.2, 96.2]	0.973 [0.926, 1.000]	0.900 [0.732, 1.000]	0.954 [0.890, 1.000]
Others	83.3 (25/30) [70.0, 96.7]	0.968 [0.880, 1.000]	0.932 [0.815, 1.000]	0.784 [0.538, 0.963]
Scanning methods				
Transvaginal	85.9 (225/262) [81.7, 90.1]	0.939 [0.902, 0.969]	0.862 [0.776, 0.935]	0.918 [0.873, 0.956]
Transabdominal	84.6 (66/78) [76.6, 92.6]	0.974 [0.938, 0.997]	0.881 [0.790, 0.953]	0.957 [0.910, 0.991]
Different centres				
Hospital A in northern region	85.6 (203/237) [81.2, 90.1]	0.966 [0.939, 0.988]	0.902 [0.853, 0.944]	0.931 [0.889, 0.966]
Hospital B in southern region	85.4 (88/103) [78.6, 92.3]	0.960 [0.923, 0.987]	0.857 [0.743, 0.949]	0.947 [0.898, 0.985]

Data in parentheses are numerators/denominators. Data in brackets are 95% confidence intervals (CIs). 95% CIs were obtained by patient-level bootstrap (5000 resamples). “Other” vendors include Samsung, Hitachi, Supersonic Imagine, and SonoScape. Hospital A in northern region = Second Affiliated Hospital of Harbin Medical University in Heilongjiang Province. Hospital B in southern region = Sichuan Provincial Maternity and Child Health Care Hospital in Sichuan Province. AUC were based on 340 and 159 patient-level predictions in the external image and video test datasets, respectively.

*AUC* Area under the receiver operating characteristic curve, *Clinical-OMTA* Clinical ovarian multi-task attention model.

^a^Videos indicate videos with expert manual correction: to ensure comparable analysis between videos and static ROI images.

### Visualisation and assessment of interpretability

The “black box” nature of DL models makes their decision-making processes difficult to interpret. To understand the focus areas of the OMTA model during the diagnostic process, we used heat map visualisation techniques for visualisation, which highlight the image regions that the model primarily focuses on when making diagnostic decisions (Fig. S6). The results of subjective assessment by an expert examiner showed that heat maps of 28.3% (103/364) and 22.4% (76/340) patients were in complete disagreement, heat maps of 12.4% (45/364) and 18.8% (64/340) patients were in partial agreement, heat maps of 21.2% (77/364) and 28.5% (97/340) patients were in majority agreement, and heat maps of 38.2% (139/364) and 30.3% (103/340) patients were in complete agreement with the subjective assessment by an expert examiner in the internal and external test datasets.

## Discussion

Accurate diagnosis of benign, borderline, and malignant adnexal masses is crucial for personalised treatment and prognosis optimisation. We developed Clinical-OMTA using datasets from mixed vendors and multiple centres to classify benign, borderline, and malignant adnexal tumours by ultrasound images, age, and CA125 data using a dual-backbone architecture (benign vs. non-benign; borderline vs. malignant). In this study, its performance was comparable to that of ADNEX and the subjective assessment by an expert examiner. When assisted by Clinical-OMTA, junior and intermediate radiologists demonstrated significantly improved diagnostic accuracy and inter-reader consistency, achieving performance comparable to that of the subjective assessment by an expert examiner. Although Clinical-OMTA supports multimodal integration, the inclusion of age and CA125 data did not enhance its performance in this study. The model maintained high performance in external test datasets for both ultrasound images and videos, across different equipment types, scanning methods, and centres. These findings indicate that Clinical-OMTA holds potential for clinical implementation.

Clinical-OMTA demonstrates good diagnostic performance. First, it enables more specific classifications of adnexal masses. Current AI models for adnexal mass evaluation primarily focus on diagnosing ovarian cancer^18,24,26,27,29–34,42–44^. Clinical-OMTA demonstrates broader diagnostic capabilities, extending to the identification of both malignant and borderline adnexal tumours, consistent with advances reported in prior deep learning systems^25,28^. The 4th edition of the WHO Classification of Female Genital Organ Tumours (2014)^4^ defined borderline adnexal tumours as “atypical hyperplasia of ovarian epithelial cells without interstitial infiltration”, discontinuing the term “low-grade malignant potential tumour”. Nevertheless, borderline adnexal tumours have ultrasound features overlapping with those of benign and malignant tumours, which can lead to misdiagnosis and potential delays or overtreatment. Clinical-OMTA first classifies masses as benign or non-benign, and then further differentiates non-benign masses into borderline or malignant categories to support clinical decision-making. Second, training two separate backbones (benign vs. non-benign; borderline vs. malignant) provided an added benefit, but the effect of the segmentation branch was architecture-dependent. As shown in Table S14, although it improved performance for the dual-backbone model, it did not confer a consistent benefit for the single-backbone model, even slightly reducing its performance. Thus, the segmentation branch does not yield a consistent performance improvement across all model architectures. Third, unlike previous studies on the classification of benign, borderline, and malignant adnexal masses^25,28^, Clinical-OMTA allows for multimodal information integration, including clinical information (age) and laboratory test index (CA125) with greyscale ultrasound features recognition to classify benign, borderline, and malignant adnexal masses for a comprehensive diagnosis. However, the binary NRI analysis showed no significant improvement of Clinical-OMTA over OMTA in either the internal or external image test datasets. This indicates that incorporating CA125 and age adds no discriminatory power to ultrasound-based models for distinguishing benign from malignant adnexal masses, which aligns with prior evidence from multiple studies^1,45,46^.

Although Clinical-OMTA shows good potential for generalisation, its robustness should be confirmed in larger, international prospective cohorts. The generalisation of AI models supports their widespread applicability in other medical centres, ensuring the clinical usability of models. Xu et al.^47^. found that classification models for thyroid tumours trained on multiple hospitals, vendors, and a national dataset exhibited the best performance during testing. Their study revealed that models trained on datasets representing the real-world data complexity and diversity can be applied to diverse sample sets, because the model can learn less-biased characteristic features from such a training dataset and enhance its generalisation. However, there is a limited number of models trained on diverse datasets in the research of adnexal masses, resulting from challenges in standardising and coordinating data from different sources. Inspired by the aforementioned studies, we incorporated heterogeneous data (from different centres and ultrasound systems) and evaluated the model on two independent external cohorts, which comprised both images and videos from two medical centres. The ROI acquisition pipeline differed between static images and videos. Although ROIs of both datasets were ultimately reviewed and finalised by expert assessment, the difference in intervention granularity (mask level vs. bounding rectangle level) may introduce hard-to-quantify effects on the image features extracted by Clinical-OMTA. Therefore, this difference somewhat limits the absolute comparability of model performance across the two acquisition modes. We also compared the fully automated and semi-automatic video pipelines, and the results demonstrated that the model also functioned effectively within a fully automated pipeline. Clinical-OMTA maintained high diagnostic performance across all external subgroups. This study included not only masses with pathological assessment results but also typical benign cases followed up by ultrasound to reduce potential issues related to the under-representation of low-risk or benign tumours. Similar to ADNEX^1^, the serum CA125 tumour marker was not a mandatory variable for establishing and validation the model’s effectiveness under conditions akin to real clinical ultrasound examinations. Thus, this study contributes to advancing the application of the adnexal mass AI model in diverse clinical settings. Notably, our video inference pipeline employs a semi-automated approach to balance efficiency and accuracy, with manual intervention as a quality control measure to correct potential segmentation errors. Future research should focus on developing fully automated methods to eliminate manual intervention while maintaining Clinical-OMTA’s diagnostic accuracy across diverse ultrasound datasets. Although we suggest that Clinical-OMTA may benefit low-resource or remote environments, 81% (276/340) of external test scans were acquired using high-end platforms (GE Voluson E8/E10, LOGIQ E9, Samsung WS80A, Mindray Resona series, Hitachi Preirus, SonoScape S60, SuperSonic Aixplorer) (Table S1), and all images were acquired and interpreted by trained radiologists. Consequently, the model’s performance with mid-range or portable ultrasound probes and by non-radiologist operators remains unknown and requires prospective evaluation in such settings. We emphasise the importance of prospective external validation of Clinical-OMTA at other centres to evaluate its generalisation, limitations, and potential for multisite deployment. Thus, the next step is to externally validate OMTA and Clinical-OMTA in a large multicentre setting, which is already underway (ChiCTR2400086850).

Clinical-OMTA has promising clinical application value. The model reduced inter-reader variability and brought the diagnostic performance of junior and intermediate radiologists closer to that of the expert. Thus, Clinical-OMTA could serve as a reliable “second opinion” for radiologists (Fig. S7). It may be integrated into ultrasound machines or used as a standalone software. With the necessary training or adjustments, radiologists can effectively use the model, thereby enabling seamless integration of AI into clinical workflows. In environments lacking radiology experts, Clinical-OMTA can assist in accurate diagnosis by reducing misdiagnoses or missed diagnoses due to inexperience and significantly improving diagnostic consistency and reliability. Additionally, the model can enhance patient outcomes through accurate and timely diagnoses and may bolster confidence in the subjective assessment by an expert examiner in challenging cases. Unlike the approach in a previous study^30^, which incorporated doctors’ diagnoses into the multimodal AI model, we adopted a strategy in which doctors used AI-assisted diagnosis. The results revealed a tiered improvement pattern, with junior radiologists deriving significantly greater benefit from AI assistance than intermediate and senior radiologists, a finding consistent with established oncology AI literature^48^. While improved inter-reader agreement demonstrates the system’s consistency, the near-perfect concordance (Fig. 3) may be indicative of automation bias, where radiologists disproportionately favoured the model’s predictions. This differential effect may stem from the inherent complexity of adnexal mass characterisation, wherein less experienced radiologists could develop over-reliance on AI guidance, especially in borderline adnexal tumours diagnosis. This raises concerns that clinicians may undervalue conflicting clinical findings and forgo second opinions in ambiguous cases. Such behaviours could compromise patient safety in real-world clinical practice. Therefore, standardising the application of AI models for the ultrasound diagnosis of adnexal masses warrants further investigation. Future studies should also incorporate longer washout periods and counterbalanced designs through randomised session orders to further reduce the risk of potential bias.

OMTA-generated heat maps highlight the ultrasound regions most influential in the model’s classification of tumours as benign, borderline, or malignant. Although heat maps have been used in previous studies^25,30^ to explain DL models for diagnosing adnexal masses, it remains unclear as to whether the areas highlighted by these maps have anatomical or pathological relevance. In this study, we conducted a preliminary investigation into the expert evaluation of the key regions identified in the heat maps. The expert judged that the heat map regions matched clinically relevant areas in 261 of 364 cases (71.7%) and 264 of 340 cases (77.6%) for the internal and external image test datasets. Conversely, the expert deemed the highlighted regions irrelevant in 103 of 364 cases (28.3%) and 76 of 340 cases (22.4%) for the internal and external image test datasets. This discordance reflects a known limitation of post-hoc interpretability methods, such as Grad-CAM, which can produce unstable or misleading visual explanations by highlighting irrelevant image regions^49,50^. Clinicians should interpret these heat maps with caution, as they do not necessarily correspond to clinically meaningful features and thus should not be relied upon for diagnostic decisions without further validation. As a purely research interpretability tool, this regional inconsistency may provide insights for future research to better understand the AI model’s decision-making process. However, the analysis methods in our study may introduce confirmation bias; independent and blinded raters would strengthen the analysis in future studies.

Our study has several limitations. First, only greyscale ultrasound images and videos were included. Although blood flow assessment is important for diagnosing adnexal masses, standardising blood flow images across different operators and machine brands is challenging. Therefore, greyscale-based AI may have more universal application value. However, we plan to further explore multimodal imaging in the future to provide more precise ultrasound diagnostic tools for adnexal masses. Additionally, videos were carefully curated for the study (2–5 s, centred ROIs, full sweep). Real-world videos differ in length, framing, and image quality. Such manual correction by experts is infeasible. Further studies should test the model in real-world scenarios. Second, all images were manually segmented for both training and testing. This pixel-wise annotation process is time-consuming and clinically impractical, which remains a significant barrier to the scalability and real-world clinical feasibility of our current approach. Future research should prioritise the development of fully automated segmentation methods to overcome this problem. Third, the study included only a single experienced ultrasound expert. However, previous literature has reported high inter-reader agreement for expert subjective assessments and ADNEX^51,52^, which helps mitigate the limitation of generalising the diagnostic performance of such assessments. Fourth, although data from multiple hospitals across China were included to enhance the model’s generalisability, the datasets still originate from a single country. Further international, multiethnic validation is still required. Additional research is needed to assess the model’s ability to conduct fine-grained classification of adnexal masses, including distinctions by clinical stage and pathological subtype. Fifth, threshold optimisation was performed on the internal test set rather than on an independent validation dataset. This introduces potential selection bias. Therefore, the reported performance on the internal test dataset should be interpreted with caution, and the primary emphasis should be placed on the external test results. Finally, this retrospective study included centres primarily focused on oncology or consultation ultrasound, leading to a higher malignancy prevalence rate than the general epidemiological data^53^. However, this is consistent with the high malignancy rates observed in other studies^24,30^, and was intended to provide a rich dataset of malignant images for thorough model training. Future research should include data from a broader range of clinical centres to optimise Clinical-OMTA.

In summary, Clinical-OMTA employs a dual-backbone architecture (benign vs. non-benign, and borderline vs. malignant) that incorporates ultrasound images, age, and CA125 to classify benign, borderline, and malignant adnexal tumours. While the framework is designed to support multimodal integration, it is important to clarify that age and CA125 did not contribute additional value to diagnostic performance in this study. Two separate backbones (benign vs. non-benign; borderline vs. malignant) of the model provide an added benefit. Its diagnostic performance was comparable to that of subjective assessment by an expert examiner and ADNEX in this study. It can improve the accuracy and consistency of junior and intermediate radiologists in classifying adnexal mass, match levels of the subjective assessment by an expert examiner, and thereby support clinical decision-making. Additionally, the model has demonstrated promising diagnostic performance across ultrasound images, video modes, different equipment vendors, scanning methods, and centres. This suggests its potential for effective implementation in diverse clinical settings. Clinical-OMTA has demonstrated good diagnostic performance, providing reliable decision-making support to radiologists and potentially enabling the integration of AI-assisted diagnosis into clinical ultrasound workflows. Future work should focus on international validation to confirm generalisability and on implementation studies that explore its integration into primary-care workflows.

## Methods

### Ethics and participants

This was a retrospective multicentre study using ultrasound and clinical datasets obtained from 23 hospitals. We collected data on patients with adnexal masses who underwent ultrasound examinations between June 2020 and May 2022 at Zhejiang Provincial People’s Hospital and the Medical Imaging Standard Database of the China National Health Commission. This dataset included data from 21 centres across 13 provinces, municipalities, and regions in China and was used for training, validation, and internal testing. The protocol was approved by the Institutional Review Board of Zhejiang Provincial People’s Hospital (Approval number QT2023117), and the requirement for written informed consent was waived. Furthermore, we collected data from ultrasound examinations at two additional hospitals, the Second Affiliated Hospital of Harbin Medical University and the Sichuan Provincial Maternity and Child Health Care Hospital, between February 2024 and April 2024 (external image test dataset) and between April 2024 and May 2024 (external video test dataset). The study protocols were approved by the Institutional Review Boards of the Second Affiliated Hospital of Harbin Medical University (KY2024-029) and the Sichuan Provincial Maternity and Child Health Care Hospital (20240205-011). All procedures involving human participants were in accordance with the ethical standards of the institutional and/or national research committees and with the principles of the 1964 Declaration of Helsinki, its later amendments, and comparable ethical standards.

Most of the present cohort has already been reported in our previous study^32^, which established a DL pipeline for ovary detection and binary classification of benign and malignant tumours. The present study extends this work by addressing the more complex task of multi-class classification of benign, borderline, and malignant tumours. To this end, we developed Clinical-OMTA, a dual-backbone architecture (benign vs. non-benign, and borderline vs. malignant) that integrates ultrasound, age, and CA125 for multi-class classification. We further evaluated its diagnostic performance in comparison with ADNEX and its generalizability across key clinical variables (e.g., acquisition modes, equipment vendors, scanning methods, centres, and histological subgroups). Inclusion and exclusion criteria were consistent across all datasets. Inclusion criteria included patients with at least one adnexal mass detected via ultrasound. All patients either underwent surgery following the ultrasound examination and obtained histopathology results (*n* = 2140) or had an ultrasound follow-up for at least 6 months until the resolution of the lesion (*n* = 241, only including corpus luteum and follicular cysts, in training, validation, and internal test datasets. All lesions in this subgroup resolved within the 6-month window. Exclusion criteria included patients with non-adnexal tumours other than metastatic ovarian tumours confirmed by histopathological analysis, as well as patients with uncertain histopathology results or without ultrasound follow-up results. The results of pathological classification of tumours were in accordance with the World Health Organization (WHO) classifications of Tumours of Female Reproductive Organs (2020)^54^. The authors (Y.N.W. and W.L.D.) had full access to and control over all data in the study with no conflicts of interest. The sample allocation and experimental design flow chart were shown in Fig. 1. The study was conducted in compliance with the STARD-2015-checklist^55^ and TRIPOD + AI-checklist^56^ (Supplementary materials checklists).

### Data collection and ultrasound examinations

All ultrasound examinations utilised International Ovarian Tumour Analysis (IOTA) terms and definitions^57^ for standardised reporting. The scanning methods (transvaginal and transabdominal) varied across different centres. Greyscale ultrasound images were obtained from image archiving and communication systems by the ultrasound departments of the participating medical centres. All ultrasound examinations were performed by radiologists (with > three years of experience) rather than sonographers and conducted on 38 different commercial ultrasound systems from nine vendors, using either transvaginal probes (5–9 MHz) or transabdominal probes (3.5–5 MHz) (Table S1). Each patient in this study was contributed only one scanning method. When a patient had multiple adnexal masses, each mass was evaluated. Typically, the target mass was stored and analysed using at least one maximal transverse image and one longitudinal image. Additional typical imaging features, such as papillary structures, posterior acoustic shadows, and solid components were included if present. Dynamic videos were recorded in greyscale at a uniform speed, with each video lasting 2–5 s. During the scan, the video was recorded from the absence of the mass to its maximum sectional view and further to its absence, to ensure a comprehensive visualisation of the whole mass. The ROI was positioned in the centre of the image to capture standardised ultrasound videos. All patients’ clinical information was obtained from electronic medical records maintained at participating medical centres. The following clinical information was recorded: age, CA125, final pathological diagnosis (including classification and subtypes). All ultrasound images, videos, and related clinical information were de-identified and encrypted at each contributing centre in accordance with institutional protocols before being securely transferred to the primary research centre, Zhejiang Provincial People’s Hospital, for quality control and analysis. The data included: (a) static images and clinical information (age and CA125) for training, validation, and testing Clinical-OMTA and OMTA; (b) dynamic videos and clinical information (age and CA125) for assessing the diagnostic performance and feasibility of Clinical-OMTA and OMTA in dynamic videos.

### Format conversion and annotation

For images, all original adnexal ultrasound images in DICOM format were anonymised and converted to PNG format (dcmtk 3.6.5, OpenCV 3.2.0). Next, the Ultrasound Image Annotation Software (version 4.4.0.1) developed by Zhejiang Demetics Medical Technology Co., Ltd was used to manually segment all adnexal masses in all image-based datasets. The manual segmentation of adnexal mass masks in images was completed by a radiologist with eight years of ultrasound experience (Y.N.W.), which was then reviewed and modified by an expert with 40 years of ultrasound experience (Y.P.B.).

### ROI extraction and standardisation

After generating the JSON annotation files, OpenCV-Python (version 4.8.1.78) was used to: (1) calculate bounding rectangles for annotated segmentation maps; (2) expand rectangles by 25 pixels in all directions; (3) crop ultrasound images to extract adnexal mass patches. Given the varying original sizes (due to multi-source ultrasound devices), all patches were resized to 352 × 352 pixels. Resizing was performed using cv2.resize with bilinear interpolation.

### Channel conversion and normalisation

To utilise pretrained parameters, all images in this study were processed as three-channel inputs. We used greyscale ultrasound images that have only a single channel. Each image was converted to three identical channels prior to normalisation. Channel-wise standardisation was then applied using mean and standard deviation values derived from previous MTANet research (Channel 1: mean = 0.485, std = 0.229; Channel 2: mean = 0.456, std = 0.224; Channel 3: mean = 0.406, std = 0.225). The same values were also used to standardise the training, validation, and testing datasets. Augmentation (Training Only): To augment training diversity and reduce overfitting, the following transformations were applied per training sample: Horizontal flip (probability = 0.5), Vertical flip (probability = 0.5), Random 90° clockwise rotation^58^. For CA125 values labelled as exceeding thresholds (e.g., “> 1000”), the threshold value (1000) was recorded. Missing CA125 values were imputed as -1 (see Table S15 for a performance comparison of imputation strategies). Before input to Clinical-OMTA, age was divided by 20 and CA125 by 30 for calibration.

### Model architecture and development

In this multicentre retrospective cohort study, we developed the OMTA using the MTANet^59,60^ for images, and further developed Clinical-OMTA by integrating clinical information (CA125 and age). The proposed Clinical-OMTA framework integrated two core components: (1) frozen MTANet-based feature extractors (MTANet was proposed in our previous study ^59^), and (2) a fully connected layer as the classification head. For comparison, OMTA was implemented using direct end-to-end probabilities without clinical information integration. The training, validation, and testing datasets comprise images with pathological labels (*n* = 2140) or ultrasound follow-up labels (*n* = 241), clinical information, radiologist-annotated segmentation masks, and algorithm-extracted ROI bounding boxes. All ultrasound images were resized and normalised prior to model development. For clinical information, missing values were imputed and standardised.

Clinical-OMTA employs dual MTANet backbones from our previous studies^32,59^ and operates in two stages (Fig. S8). Stage 1 trained two MTANet backbones: one to separate benign from non-benign masses (MTANet_BvNB) and another to distinguish malignant from borderline lesions (MTANet_MvB). Both were trained with a hybrid classification-segmentation loss (MTANet_MvB used only the malignant/borderline subset). The segmentation branch allowed the backbone networks to achieve better classification performance (Table S14). Each backbone outputs per-image class probabilities, where ${{\boldsymbol{p}}}_{{\boldsymbol{Benign}}}$ is the benign-class probability from MTANet_BvNB, and ${{\boldsymbol{p}}}_{{\boldsymbol{Malignant}}}$ is the malignant-class probability from MTANet_MvB. The predicted probabilities of borderline and malignant adnexal tumours in Stage 1 are computed as:

1 $${\hat{{\boldsymbol{p}}}}_{{\boldsymbol{Borderline}}}={({\bf{1}}-{\boldsymbol{p}}}_{{\boldsymbol{Benign}}})\,\cdot \,({\bf{1}}-{{\boldsymbol{p}}}_{{\boldsymbol{Malignant}}\,})$$

2 $${\hat{{\boldsymbol{p}}}}_{{\boldsymbol{Malig}}{\boldsymbol{n}}{\boldsymbol{ant}}}={({\bf{1}}-{\boldsymbol{p}}}_{{\boldsymbol{Benign}}})\,\cdot \,{{\boldsymbol{p}}}_{{\boldsymbol{Malignant}}\,}$$

In Stage 2, we concatenated (i) MTANet_BvNB’s benign-class probability ${{\boldsymbol{p}}}_{{\boldsymbol{Benign}}}$; (ii) the two additional probabilities ${\hat{{\boldsymbol{p}}}}_{{\boldsymbol{Borderline}}}$ and ${\hat{{\boldsymbol{p}}}}_{{\boldsymbol{Malignant}}}$; and (iii) normalised age and CA125, yielding a five-dimensional input to the classification head. The classification head then output a three-dimensional probability vector (${{\boldsymbol{P}}}_{{\boldsymbol{Benign}}}$, ${{\boldsymbol{P}}}_{{\boldsymbol{Borderline}}}$, and ${{\boldsymbol{P}}}_{{\boldsymbol{Malig}}{\boldsymbol{n}}{\boldsymbol{ant}}}$) for the given five-dimensional input. We trained and assessed different classification heads including fully connected layer via PyTorch, and machine learning based classification heads via TPOT (version 0.12.2) and Scikit-learn (version 1.4.1.post1) (Table S15). During the training of these classification heads, we froze the backbone parameters and optimised only the classification heads using the training dataset feature vectors. After comparing performance on the training and validation dataset, we selected the fully connected layer as the default configuration for both Clinical-OMTA and OMTA models. Our assessment of different classification heads on the external image test dataset also showed no significant differences (Table S15).

### Prediction and thresholding

Clinical-OMTA outputs per-image probabilities for benign, borderline, and malignant classes. Patient-level predictions aggregated probabilities from multiple images via the mean probability. Final diagnoses used tiered thresholds: Benign:${{\boldsymbol{P}}}_{{\boldsymbol{Benign}}}$ > 0.65; Borderline: ${{\boldsymbol{P}}}_{{\boldsymbol{Benign}}}$ ≤ 0.65 and ${{\boldsymbol{P}}}_{{\boldsymbol{Borderline}}}$ > 0.15; Malignant: Otherwise. This hierarchical approach replaces direct argmax selection. Thresholds were empirically optimised on the internal test dataset to maximise the sum of sensitivity and specificity and could be adjusted for other populations^61,62^.

### Implementation details

Models were implemented using PyTorch and trained on an NVIDIA RTX 3080 GPU. The training configuration employed a batch size of 10 and the AdamW optimiser with an initial learning rate of 10^-^⁵. Training proceeded for 200 epochs, with the learning rate decaying by a factor of 0.1 every 30 epochs. We adopted the MTANet architecture, which was built upon the PVTv2-b2 framework^60^ and incorporated additional custom layers and modules for multi-task learning. For initialisation, only the PVTv2-b2 components within MTANet were loaded with weights pre-trained on ImageNet-1k; all newly introduced layers of MTANet were randomly initialised. In the Clinical-OMTA model, the classification head was also randomly initialised and trained separately from the MTANet backbones. We randomly withheld 10% of the training images as a validation subset for hyperparameter tuning.

### Model performance and comparison to ADNEX and subjective assessment by an expert examiner

The performance of OMTA and Clinical-OMTA was evaluated and compared with that of subjective assessment by an expert examiner using the ADNEX model (10% risk threshold for malignant tumours as recommended by the European consensus statement)^63^ in a mobile application.

### Performance and inter-reader agreement of Clinical-OMTA-assisted radiologists of different seniority levels

The diagnostic performance of 11 ultrasound radiologists, including one expert (40 years of ultrasound experience), four intermediate radiologists (5–10 years of ultrasound experience), and six junior radiologists (3–5 years of ultrasound experience), was compared with that of Clinical-OMTA-assisted evaluation. All radiologists independently evaluated the ultrasound images of 704 patients (from the internal and external image test datasets) combined with age and CA125 data. They were blinded to the clinical history, original clinical records, and pathological results, and independently assessed all anonymised and randomised lesions. They first performed an independent assessment at the patient level and predicted whether adnexal masses were benign, borderline, or malignant. With AI assistance, radiologists performed a second evaluation of ultrasound images of patients in the same test dataset after a two-week washout period^47^ and predicted whether adnexal masses were benign, borderline, or malignant. Results of Clinical-OMTA provided to radiologists included classification results, visualised heat maps of each image, and per-image probabilities of benign, borderline, or malignant adnexal mass on each image determined by Clinical-OMTA. The levels of inter-reader agreement (using the Kappa coefficient) among different radiologist groups, both with and without Clinical-OMTA assistance, were compared.

### Generalising the model to different scenarios

We evaluated the feasibility of applying a static image-based diagnostic model to ultrasound videos. To ensure comparable analysis between two acquisition modes, all ROIs conform to the same expert extraction standard, meaning that the same expert reviewed and finalised every ROI. However, the specific process differed. For static images, manual segmentation of adnexal mass masks in all images was completed by a radiologist (Y.N.W.), which was then reviewed and modified by the expert. Then, ROI bounding rectangles were generated for identified tumours. For videos, given fully manual processing of all video frames was time-consuming, we adopted a semi-automated approach (Table S11). First, a U-Net-like segmentation model^32^, trained on the annotated training image dataset, was used to preliminarily extract target tumour masks from videos. Then, the bounding rectangles of these tumours were generated according to these tumour masks and expanded by 25 pixels frame by frame. But the automatically generated frame-by-frame bounding rectangles may contain errors. To ensure the performance comparability between video and static image analyses, we adopted the expert correction step. This step ensured that every video frame’s ROI was defined by an appropriately sized bounding rectangle fully enclosing the tumour, thereby correcting segmentation errors such as under-segmentation, over-segmentation, misplacement, or multi-segmentation. The number of corrected frames per video is shown in Fig. S9. In cases of multi-segmentation, the expert resolved the ambiguity by visually selecting the single most clinically relevant region and manually adjusting the detection box to encompass it. This process ensured that a single, unified detection box was defined for each frame prior to cropping. Subsequently, the ROI for each video frame was cropped and normalised according to the final, expert-selected and adjusted detection box, then input into the Clinical-OMTA model for frame-by-frame analysis. Clinical-OMTA independently processes each frame, calculating their probability values.

The Clinical-OMTA model first performed a complete inference on each video frame independently. Each frame’s five-dimensional fused feature vector was processed by the pre-trained fully connected layer to generate a frame-wise, three-dimensional probability vector. These individual probability vectors were then aggregated across all frames and averaged to produce a single video-level probability. Finally, based on the hierarchical threshold criteria, this averaged probability was used to output the diagnostic classification for the entire video.

This experiment was designed to evaluate the performance of the Clinical-OMTA model in distinguishing between benign, borderline, and malignant adnexal tumours in videos. In practical applications, the initial ROI extraction step, including box correction, can be fully automated by optimising the segmentation model. For detailed methodology regarding tumour segmentation model construction, please refer to our previous study^32^. We also analysed the model’s performance in different subgroups of the external test cohort, including imaging modes (images and videos), various equipment vendors (GE, Philips, Mindray, and others), scanning methods (transvaginal and transabdominal), different centres (grouped into northern and southern regions of China), and histological subgroups (germ cell, serous, mucinous, sex cord stromal, mixed, and other tumours).

### Model visualisation and interpretability assessment

Heat maps generated using Gradient-weighted Class Activation Mapping (Grad-CAM) were used to visualise key areas of ultrasound features employed by the OMTA for diagnosis on the internal and external test datasets^64^. If the OMTA model classifies a mass as benign, the heat map is generated from the benign/non-benign MTANet, highlighting the region that supports benign classification. If a mass is classified as borderline, the heat map is generated from the borderline/malignant MTANet, showing the region relevant to borderline classification. Similarly, if a mass is classified as malignant, the heat map is also generated from the borderline/malignant MTANet, indicating the regions that contribute to malignant classification. The expert evaluated the key regions identified in the heat maps. The expert’s own image interpretation served as the reference standard for the internal and external test datasets, and the same expert subjectively interpreted and scored the heat maps as follows: complete disagreement, 0 point; partial agreement, 1 point; mostly agreement, 2 points; complete agreement, 3 points.

### Statistical analysis

The diagnostic performance of OMTA, Clinical-OMTA, and the ADNEX model was assessed by constructing receiver operating characteristic curves and calculating the corresponding AUCs. Differences in AUCs were evaluated using DeLong’s test. The 95% CIs for sensitivity, specificity, accuracy, Cohen’s kappa, and F1-score of the diagnoses made by OMTA, Clinical-OMTA, ADNEX, and an expert examiner were computed using the non-parametric bootstrap percentile method (with 5000 resamples)^24^. The accuracy of probability predictions, reflecting model calibration, was quantified using the Brier score. The McNemar test was employed for pairwise comparisons of diagnostic accuracy. NRI^41^ was applied to quantify the incremental value of incorporating age and CA125 into the image-only model. All analyses were performed using Python (version 3.10.12), utilising the Scikit-learn (version 1.4.1.post1) and SciPy (version 1.11.2) packages, with a two-sided *P*-value < 0.05 considered statistically significant.

## Supplementary information

Supplementary Material File

## Data Availability

As the study involved human participants, the data cannot be made freely available in the manuscript or a public repository due to ethical restrictions. However, the data are available from Zhejiang Provincial People’s Hospital to researchers who meet the criteria for accessing confidential data. Interested researchers may send data access requests to the corresponding author (L.T.S.). De-identified DICOM images, clinical variables, and model code will be provided for non-commercial research upon receipt of a data-use agreement and local IRB approval. Requests should be emailed to sunlitao@hmc.edu.cn and will be reviewed by the Ethics Committee of Zhejiang Provincial People’s Hospital.

## Code availability

The codes of this paper are available at https://github.com/SLYXDWL/OvaMTA. It should only be used for academic research.
