# Supplementary material for: Development and validation of an artificial intelligence-based model for diagnosing benign, borderline, and malignant adnexal masses
Source: NPJ Precis Oncol. 2026 Feb 3;10:106. doi: 10.1038/s41698-026-01320-5 (PMC12976284; doi:10.1038/s41698-026-01320-5)
Supplement: Supplementary file 1 — Supplementary Material File [file 41698_2026_1320_MOESM1_ESM.docx]

Supplementary Materials

Tables

Table S1: Vendor distribution.

| Vendor | Type | Training and validation dataset  (n = 1,518) | | Internal test dataset  (n = 364) | External image test dataset  (n = 340) | External video test dataset  (n = 159) |
| --- | --- | --- | --- | --- | --- | --- |
| GE  (n = 1,570) |  | | 989 (66.2) | 263 (72.3) | 214 (62.9) | 102 (64.2) |
|  | Voluson E8 | | 552 (36.4) | 152 (41.8) | 197 (57.4) | 37 (23.3) |
|  | Voluson E10 | | 196 (12.9) | 44 (12.1) | 15 (4.1) | 21 (13.2) |
|  | Voluson E6 | | 62 (4.1) | 23 (6.3) | — | 13 (8.2) |
|  | LOGIQ E9 | | 89 (5.9) | 29 (8.0) | 2 (0.6) | 26 (16.4) |
|  | LOGIQ E8 | | 4 (0.3) | — | — | — |
|  | LOGIQ 5PRO | | 3 (0.2) | 2 (0.6) | — | — |
|  | Voluson 730 | | 61 (4.0) | 2 (0.6) | — | 2 (1.3) |
|  | S7 | | 1 (0.1) | — | — | — |
|  | S10 | | 21 (1.4) | 11 (3.0) | — | 5 (3.1) |
| Samsung  (n = 385) |  | | 277 (18.3) | 59 (16.2) | 10 (2.9) | 39 (24.5) |
|  | WS80A | | 233 (15.4) | 45 (12.4) | 3 (0.9) | 28 (17.6) |
|  | RS80A | | 37 (2.4) | 11 (3.0) | — | 8 (5.0) |
|  | HS70A | | 3 (0.2) | — | — | — |
|  | HERA XW10 | | 2 (0.1) | 3 (0.8) | — | 3 (1.9) |
|  | HERA | | 2 (0.1) | — | — | — |
|  | MedisionV20-2 | | — | — | 1 (0.3) | — |
|  | Medision-4 | | — | — | 2 (0.6) | — |
|  | W102 | | — | — | 1 (0.3) | — |
|  | W10A | | — | — | 3 (0.9) | — |
| Philips  (n = 291) |  | | 186 (12.3) | 33 (9.1) | 57 (16.8) | 15 (9.4) |
|  | IU22 | | 30 (2.0) | 3 (0.8) | — | 3 (1.9) |
|  | HD 15 | | 17 (1.1) | 3 (0.8) | 12 (3.5) | — |
|  | EPIQ7 | | 31 (2.0) | 7 (1.9) | — | 5 (3.1) |
|  | Affiniti50 | | 57 (3.8) | 9 (2.5) | — | 7 (4.4) |
|  | IU Elite | | 15 (1.0) | 1 (0.3) | — | — |
|  | EPIQ5 | | 13 (0.9) | — | — | — |
|  | Affiniti70 | | 23 (1.5) | 10 (2.8) | 45 (13.2) | — |
| Mindray  (n = 80) |  | | 36 (2.37) | 4 (1.1) | 39 (10.7) | 1 (0.6) |
|  | Resona 7 | | 5 (0.3) | 1 (0.3) | 2 (0.6) | — |
|  | DC-8 | | 2 (0.1) | — | — | — |
|  | Resona R7S | | — | — | 1 (0.3) | — |
|  | Resona 8S | | — | — | 2 (0.6) | — |
|  | Resona 8 | | 22 (1. 5) | 2 (0.6) | 34 (10.0) | — |
|  | Resona 9 | | 7 (0.5) | 1 (0.3) | — | 1 (0.6) |
| HITACHI  (n = 33) | Preirus | | 18 (1.2) | 4 (1.1) | 11 (3.2) | — |
| SonoScape  (n = 13) |  | | 5 (0.3) | — | 8 (2.4) | — |
|  | S60 | | 3 (0.2) | — | 8 (2.4) | — |
|  | S50 | | 2 (0.1) | — | — | — |
| SIEMENS  (n = 5) |  | | 4 (0.3) | 1 (0.3) | — | — |
|  | ACUSON Sequoia | | 2 (0.1) | 1 (0.3) | — | — |
|  | S3000 | | 2 (0.1) | — | — | — |
| Toshiba  (n = 2) | Aplio500 | | 2 (0.1) | — | — | — |
| SuperSonic Imagine  (n =2) | Aixplorer  Ultimate | | 1 (0.1) | — | 1 (0.3) | — |

Note. — Data are numbers of patients with percentages in parentheses.Table S2. Diagnostic performance of OMTA, Clinical-OMTA, ADNEX, and the expert examiner for classifying benign, borderline and malignant adnexal tumours in the internal test dataset.

| Mode | Classification | AUC | SEN (%) | SPE (%) | ACC (%) | Kappa | | F1-score (%) |
| --- | --- | --- | --- | --- | --- | --- | --- | --- |
| OMTA | Benign | 0.941  [0.931, 0.956] | 85.2 (156/183)  [80.1, 90.2] | 88.4 (160/181)  [83.6, 90.2] | 79.4 (289/364)  [75.3, 83.5] | | 0.745  [0.674, 0.805] | 79.6  [75.2, 83.5] |
|  | Borderline | 0.849  [0.823, 0.896] | 53.8 (28/52)  [40.4, 67.3] | 92.3 (288/312)  [89.2, 95.2] |  |  |  |  |
|  | Malignant | 0.940  [0.924, 0.958] | 81.4 (105/129)  [74.4, 87.9] | 87.2 (205/235)  [82.8, 91.4] |  |  |  |  |
| Clinical-OMTA | Benign | 0.938  [0.924, 0.955] | 86.9 (159/183)  [82.0, 91.6] | 87.3 (158/181)  [82.2, 91.9] | 79.4 (289/364)  [75.0, 83.5] | | 0.769  [0.750, 0.826] | 79.4  [74.9, 83.4] |
|  | Borderline | 0.839  [0.808, 0.884] | 48.1 (25/52)  [34.6, 61.5] | 91.7 (286/312)  [88.5, 94.6] |  |  |  |  |
|  | Malignant | 0.941  [0.929, 0.955] | 81.4 (105/129)  [74.4, 87.9] | 88.9 (209/235)  [84.7, 92.8] |  |  |  |  |
| ADNEX | Benign | 0.942  [0.925, 0.960] | 81.4 (149/183)  [75.4, 86.9] | 94.5 (171/181)  [90.9, 97.7] | 79.4 (289/364)  [75.0, 83.5] | | 0.740  [0.673, 0.801] | 78.9  [74.4, 83.2] |
|  | Borderline | 0.868  [0.841, 0.896] | 40.4 (21/52)  [26.8, 53.7] | 95.8 (299/312) [93.5, 98.0] |  |  |  |  |
|  | Malignant | 0.910  [0.892, 0.925] | 92.2 (119/129)  [87.2, 96.7] | 77.9 (183/235)  [72.4, 83.1] |  |  |  |  |
| Expert | Benign | — | 85.2 (156/183) [79.8, 90.3] | 86.2 (156/181) [80.9, 90.9] | 81.6 (297/364) [77.5, 85.4] | | 0.775  [0.705, 0.832] | 81.2  [76.8, 85.1] |
|  | Borderline | — | 50.0 (26/52) [36.4, 63.4] | 94.9 (296/312) [92.2, 97.1] |  |  |  |  |
|  | Malignant | — | 89.1 (115/129) [83.6, 94.2] | 88.9 (209/235) [84.6, 92.7] |  |  |  |  |

**Note.** — Data in parentheses are numerators/denominators; data in brackets are 95% confidence intervals (CIs). 95% CIs were obtained by patient-level bootstrap (5,000 resamples). AUC = Area under the receiver operating characteristic curve, SPE = Specificity, SEN = Sensitivity, ACC = Accuracy, OMTA = Ovarian multi-task attention model, Clinical-OMTA = Clinical ovarian multi-task attention model, ADNEX = Assessment of Different NEoplasias in the adneXa. **Table S3.** **Diagnostic performance of OMTA, Clinical-OMTA, ADNEX, and the expert examiner for classifying benign, borderline and malignant adnexal tumours in the external image test dataset.**

| Mode | Classification | AUC | SEN (%) | SPE (%) | ACC (%) | Kappa | F1-score (%) |
| --- | --- | --- | --- | --- | --- | --- | --- |
| OMTA | Benign | 0.943  [0.929, 0.963] | 90.7 (205/226) [86.8, 94.3] | 85.1 (97/114)  [78.4, 91.3] | 83.5 (284/340)  [79.4, 87.4] | 0.760  [0.684, 0.827] | 83.6  [79.5, 87.4] |
|  | Borderline | 0.834  [0.796, 0.875] | 51.4 (19/37)  [35.5, 67.6] | 94.1 (285/303)  [91.3, 96.7] |  |  |  |
|  | Malignant | 0.919  [0.898, 0.944] | 77.9 (60/77)  [68.2, 86.8] | 92.0 (242/263)  [88.7, 95.0] |  |  |  |
| Clinical-OMTA | Benign | 0.950  [0.938, 0.962] | 93.8 (212/226)  [90.5, 96.7] | 85.1 (97/114)  [78.4, 91.3] | 85.6 (291/340)  [81.8, 89.1] | 0.803  [0.735, 0.864] | 85.4  [81.4, 89.1] |
|  | Borderline | 0.870  [0.842, 0.902] | 48.7 (18/37)  [32.4, 64.7] | 95.1 (288/303)  [92.5, 97.4] |  |  |  |
|  | Malignant | 0.930  [0.913, 0.948] | 79.2 (61/77)  [69.7, 88.0] | 93.5 (246/263)  [90.5, 96.3] |  |  |  |
| ADNEX | Benign | 0.953  [0.942, 0.966] | 92.9 (210/226)  [89.3, 96.1] | 83.3 (95/114)  [76.4, 89.9] | 84.1 (286/340)  [80.0, 87.9] | 0.774  [0.699, 0.842] | 82.4  [77.7, 86.8] |
|  | Borderline | 0.853  [0.806, 0.901] | 21.6 (8/37)  [9.1, 35.3] | 98.7 (299/303)  [97.3, 99.7] |  |  |  |
|  | Malignant | 0.938  [0.917, 0.954] | 88.3 (68/77)  [80.7, 95.2] | 88.2 (232/263)  [84.1, 92.1] |  |  |  |
| Expert | Benign | — | 96.0 (217/226) [93.3, 98.3] | 81.6 (13/37) [74.0, 88.3] | 87.4 (297/340) [83.5, 90.6] | 0.821  [0.754, 0.878] | 86.2  [81.9, 90.0] |
|  | Borderline | — | 35.1 (13/37) [20.0, 51.3] | 98.7 (299/303) [97.3, 99.7] |  |  |  |
|  | Malignant | — | 87.0 (67/77) [79.2, 94.1] | 93.2 (245/263) [90.0, 96.1] |  |  |  |

Note. — Data in parentheses are numerators/denominators; data in brackets are 95% confidence intervals (CIs). 95% CIs were obtained by patient-level bootstrap (5,000 resamples). AUC = Area under the receiver operating characteristic curve, SPE = Specificity, SEN = Sensitivity, ACC = Accuracy, OMTA = Ovarian multi-task attention model, Clinical-OMTA = Clinical ovarian multi-task attention model, ADNEX = Assessment of Different NEoplasias in the adneXa. Table S4. Mean performance of 11 radiologists’ subjective assessments assisted with and without Clinical-OMTA in the internal and external image test datasets.

| Mode | Datasets | Classification | SEN (%) | SPE (%) | ACC (%) | Kappa | F1-score (%) |
| --- | --- | --- | --- | --- | --- | --- | --- |
| Radiologist assessment | Internal test | Benign | 81.4 [79.8, 82.9] | 74.3 [72.5, 76.2] | 69.8 [68.4, 71.3] | 0.596  [0.573, 0.618] | 68.4  [67.0, 69.9] |
|  |  | Borderline | 33.2 [29.6, 36.6] | 90.6 [89.6, 91.5] |  |  |  |
|  |  | Malignant | 68.2 [65.9, 70.5] | 85.5 [84.3, 86.7] |  |  |  |
|  | External test | Benign | 80.7 [79.3, 82.1] | 72.3 [69.8, 74.6] | 72.3 [70.9, 73.7] | 0.567  [0.542, 0.593] | 71.7  [70.3, 73.2] |
|  |  | Borderline | 23.6 [19.6, 27.6] | 93.0 [92.2, 93.9] |  |  |  |
|  |  | Malignant | 71.1 [68.2, 74.0] | 84.3 [83.0, 85.5] |  |  |  |
| Radiologist assessment assisted with Clinical-OMTA | Internal test | Benign | 91.1 [89.8, 92.3] | 88.4 [87.0, 89.8] | 84.5[83.4, 85.6] | 0.828  [0.812, 0.844] | 84.1  [83.0, 85.3] |
|  |  | Borderline | 54.1 [50.1, 58.2] | 94.8 [94.0, 95.5] |  |  |  |
|  |  | Malignant | 87.4 [85.7, 89.1] | 91.8 [90.7, 92.8] |  |  |  |
|  | External test | Benign | 96.4 [95.7, 97.1] | 85.0 [82.8, 86.8] | 88.0 [86.9, 89.0] | 0.835  [0.818, 0.853] | 87.4  [86.3, 88.5] |
|  |  | Borderline | 47.2 [42.2, 52.2] | 97.1 [96.5, 97.7] |  |  |  |
|  |  | Malignant | 82.8 [80.3, 85.2] | 94.4 [93.5, 95.3] |  |  |  |

Note. — Data in brackets are 95% confidence intervals (CIs). 95% CIs for reader-averaged performance were obtained via patient-level bootstrap (5,000 resamples), wherein within each resample the metric was calculated per reader and then averaged across all readers. The CIs were conditional on the fixed panel of readers. SPE = Specificity, SEN = Sensitivity, ACC = Accuracy.

Table S5. Radiologists’ subjective assessments of ultrasound images and clinical information in the internal test dataset.

| Readers | Classification | SEN (%) | SPE (%) | ACC (%) | Kappa | F1-score (%) |
| --- | --- | --- | --- | --- | --- | --- |
| Junior radiologists | | | | | | |
| Reader 1 | Benign | 70.5 (129/183) [63.8, 76.9] | 82.9 (150/181) [77.1, 88.1] | 68.4 (249/364) [63.5, 73.1] | 0.616 [0.536, 0.692] | 69.8 [65.1, 74.2] |
|  | Borderline | 53.8 (28 /52) [40.0, 67.3] | 82.4 (257/312) [77.9, 86.5] |  |  |  |
|  | Malignant | 71.3 (92/129) [63.2, 78.8] | 87.7 (206/235) [83.4, 91.7] |  |  |  |
| Reader 2 | Benign | 88.0 (161/183) [82.9, 92.4] | 58.0 (105/181) [50.8, 65.2] | 68.1 (248/364) [63.2, 72.8] | 0.521 [0.430, 0.606] | 62.6 [57.0, 68.0] |
|  | Borderline | 0.0 (0/52) [0.0, 0.0] | 100 (312/312) [100, 100] |  |  |  |
|  | Malignant | 67.4 (87/ 129) [59.1, 75.6] | 83.0 (195/235) [78.0, 87.5] |  |  |  |
| Reader 3 | Benign | 83.6 (153/ 183) [78.1, 88.7] | 74.0 (134/181) [67.5, 80.2] | 69.5 (253/364) [64.6, 74.2] | 0.639 [0.563, 0.710] | 66.0 [60.6, 71.2] |
|  | Borderline | 0.0 (0/52) [0.0, 0.0] | 93.9 (293/312) [91.1, 96.4] |  |  |  |
|  | Malignant | 77.5 (100/129) [70.1, 84.8] | 80.9 (190/235) [75.8, 85.7] |  |  |  |
| Reader 4 | Benign | 95.6 (175/183) [92.5, 98.3] | 55.8 (101/181) [48.4, 62.8] | 62.4 (227/364) [57.4, 67.3] | 0.494 [0.414, 0.572] | 59.2 [53.4, 65.0] |
|  | Borderline | 28.8 (15/52) [16.7, 41.3] | 82.7 (258/312) [78.6, 86.8] |  |  |  |
|  | Malignant | 28.7 (37/ 129) [21.0, 37.0] | 98.7 (232/235) [97.0, 100] |  |  |  |
| Reader 5 | Benign | 90.7 (166/ 183) [86.3, 94.7] | 62.4 (113/181) [55.3, 69.6] | 70.6 (257/364) [65.9, 75.3] | 0.568 [0.484, 0.649] | 69.4 [64.4, 74.5] |
|  | Borderline | 36.5 (19/52) [23.2, 50.0] | 92.0 (287/312) [88.9, 94.9] |  |  |  |
|  | Malignant | 55.8 (72/129) [47.2, 64.7] | 94.0 (221/235) [90.8, 97.0] |  |  |  |
| Reader 6 | Benign | 37.2 (68/183) [30.4, 44.4] | 88.4 (160/181) [83.6, 92.7] | 52.5 (191/364) [47.3, 57.4] | 0.278 [0.201, 0.354] | 49.9 [44.3, 55.2] |
|  | Borderline | 17.3 (9/52) [7.5, 28.1] | 93.3 (291/312) [90.3, 95.9] |  |  |  |
|  | Malignant | 88.4 (114/129) [82.4, 93.7] | 44.3 (104/235) [37.8, 50.6] |  |  |  |
| Intermediate radiologists | | | | | | |
| Reader 7 | Benign | 91.3 (167/183) [86.9, 95.1] | 69.1 (125/181) [62.2, 75.7] | 73.1 (266/364) [68.4, 77.5] | 0.648 [0.568, 0.719] | 71.8 [66.8, 76.6] |
|  | Borderline | 28.8 (15/52) [17.1, 41.5] | 92.6 (289/312) [89.6, 95.4] |  |  |  |
|  | Malignant | 65.1 (84/129) [56.6, 73.3] | 91.9 (216/235) [88.4, 95.2] |  |  |  |
| Reader 8 | Benign | 68.3 (125/183) [61.3, 75.1] | 96.1 (174/181) [93.1, 98.8] | 68.7 (250/364) [64.0, 73.4] | 0.702 [0.635, 0.761] | 71.3 [66.8, 75.6] |
|  | Borderline | 50.0 (26/52) [36.5, 64.0] | 78.2 (244/312) [73.6, 82.8] |  |  |  |
|  | Malignant | 76.7 (99/129) [69.1, 83.7] | 83.4 (196/235) [78.6, 88.0] |  |  |  |
| Reader 9 | Benign | 92.3 (169/183) [88.3, 96.0] | 65.2 (118/181) [58.3, 72.1] | 73.6 (268/364) [69.2, 78.0] | 0.584 [0.499, 0.662] | 72.7 [67.9, 77.5] |
|  | Borderline | 55.8 (29/52) [42.0, 69.4] | 92.0 (287/312) [88.9, 94.8] |  |  |  |
|  | Malignant | 54.3 (70/129) [45.6, 62.9] | 96.6 (227/235) [94.2, 98.7] |  |  |  |
| Reader 10 | Benign | 92.3 (169/183) [88.3, 95.9] | 79.6 (144/181) [73.5, 85.3] | 79.7 (290/364) [75.5, 83.8] | 0.741 [0.672, 0.805] | 78.9 [74.4, 83.3] |
|  | Borderline | 44.2 (23/52) [30.4, 58.0] | 94.6 (295/312) [91.9, 96.9] |  |  |  |
|  | Malignant | 76.0 (98/129) [68.3, 83.2] | 91.5 (215/235) [87.8, 94.8] |  |  |  |
| The expert examiner | | | | | | |
| Reader 11 | Benign | 85.2 (156/183) [79.8, 90.3] | 86.2 (156/181) [80.9, 90.9] | 81.6 (297/364) [77.5, 85.4] | 0.775 [0.705, 0.832] | 81.2 [76.8, 85.1] |
|  | Borderline | 50.0 (26/52) [36.4, 63.4] | 94.9 (296/312) [92.2, 97.1] |  |  |  |
|  | Malignant | 89.1 (115/129) [83.6, 94.2] | 88.9 (209/235) [84.6, 92.7] |  |  |  |

Note. — Data in parentheses are numerators/denominators; data in brackets are 95% confidence intervals (CIs). 95% CIs were obtained by patient-level bootstrap (5,000 resamples). SPE = Specificity, SEN = Sensitivity, ACC = Accuracy.

Table S6. Clinical-OMTA-assisted evaluation of ultrasound images and clinical information in the internal test dataset.

| Readers | Classification | SEN (%) | SPE (%) | ACC (%) | Kappa | F1-score (%) |
| --- | --- | --- | --- | --- | --- | --- |
| Junior radiologists | | | | | | |
| Reader 1 | Benign | 89.1 (163/183) [84.4, 93.3] | 87.8 (159/181) [82.9, 92.3] | 83.8 (305/364) [79.9, 87.4] | 0.818 [0.759, 0.869] | 83.3 [79.1, 89.1] |
|  | Borderline | 50.0 (26/52) [36.4, 63.8] | 95.2 (297/312) [92.7, 97.4] |  |  |  |
|  | Malignant | 89.9 (116/129) [84.4, 94.8] | 90.6 (213/235) [86.7, 94.1] |  |  |  |
| Reader 2 | Benign | 88.0 (161/183) [83.2, 92.4] | 86.7 (157/181) [81.4, 91.4] | 79.9 (291/364) [75.8, 83.8] | 0.776 [0.713, 0.832] | 79.9 [75.6, 83.9] |
|  | Borderline | 50.0 (26/52) [36.2, 63.6] | 91.7 (286/312) [88.5, 94.6] |  |  |  |
|  | Malignant | 80.6 (104/129) [73.6, 87.2] | 90.2 (212/235) [86.1, 93.8] |  |  |  |
| Reader 3 | Benign | 91.3 (167/183) [87.0, 95.0] | 86.2 (156/181) [80.8, 91.0] | 84.6 (308/364) [80.8, 88.2] | 0.827 [0.768, 0.877] | 84.1 [79.8, 87.8] |
|  | Borderline | 50.0 (26/52) [36.2, 63.6] | 95.8 (299/312) [93.5, 98.0] |  |  |  |
|  | Malignant | 89.1 (115/129) [83.3, 94.2] | 92.3 (217/235) [88.8, 95.5] |  |  |  |
| Reader 4 | Benign | 92.9 (170/183) [89.1, 96.3] | 88.4 (160/181) [83.5, 92.9] | 84.3 (307/364) [80.5, 87.9] | 0.836 [0.782, 0.883] | 84.2 [80.3, 87.8] |
|  | Borderline | 57.7 (30/52) [44.2, 70.8] | 93.3 (291/312) [90.4, 95.9] |  |  |  |
|  | Malignant | 82.9 (107/129) [76.1, 89.2] | 93.6 (220/235) [90.3, 96.5] |  |  |  |
| Reader 5 | Benign | 91.8 (168/183) [87.6, 95.6] | 87.8 (159/181) [83.0, 92.4] | 84.3(307/364) [80.2, 87.9] | 0.834 [0.777, 0.881] | 84.0 [79.8, 87.7] |
|  | Borderline | 51.9 (27/52) [38.1, 65.4] | 94.6 (295/312) [91.9, 96.9] |  |  |  |
|  | Malignant | 86.8 (112/129) [80.5, 92.3] | 92.3 (217/235) [88.7, 95.5] |  |  |  |
| Reader 6 | Benign | 87.4 (160/183) [82.6, 92.0] | 86.2 (156/181) [80.9, 91.0] | 80.5 (293/364) [76.4, 84.3] | 0.781 [0.717, 0.836] | 79.7 [75.0, 83.8] |
|  | Borderline | 38.5 (20/52) [25.5, 52.0] | 94.6 (295/312) [91.9, 97.0] |  |  |  |
|  | Malignant | 87.6 (113/129) [81.5, 93.0] | 87.7 (206/235) [83.3, 91.7] |  |  |  |
| Intermediate radiologists | | | | | | |
| Reader 7 | Benign | 92.3 (169/183) [88.4, 96.0] | 87.3 (158/181) [82.3, 91.9] | 83.8 (305/364) [79.9, 87.4] | 0.826 [0.769, 0.876] | 83.4 [79.3, 87.1] |
|  | Borderline | 50.0 (26/52) [36.7, 63.0] | 94.6 (295/312) [91.9, 97.0] |  |  |  |
|  | Malignant | 85.3 (110/129) [78.8, 91.1] | 91.9 (216/235) [88.4, 95.2] |  |  |  |
| Reader 8 | Benign | 88.5 (162/183) [83.8, 92.9] | 92.3 (167/181) [88.2, 96.0] | 86.0 (313/364) [82.4, 89.3] | 0.828 [0.768, 0.879] | 86.0 [82.4, 89.4] |
|  | Borderline | 69.2 (36/52) [56.4, 81.1] | 94.6 (295/312) [91.9, 96.9] |  |  |  |
|  | Malignant | 89.1 (115/129) [83.5, 94.2] | 91.5 (215/235) [87.9, 94.8] |  |  |  |
| Reader 9 | Benign | 93.4 (171/183) [89.7, 96.7] | 90.6 (164/181) [86.0, 94.6] | 87.4 (318/364) [83.8, 90.7] | 0.867 [0.818, 0.909] | 87.0 [83.2, 90.4] |
|  | Borderline | 57.7 (30/52) [43.8, 70.5] | 96.2 (300/312) [93.8, 98.1] |  |  |  |
|  | Malignant | 90.7 (117/129) [85.3, 95.5] | 92.8 (218/235) [89.2, 95.8] |  |  |  |
| Reader 10 | Benign | 95.1 (174/183) [91.7, 97.9] | 90.1 (163/181) [85.5, 94.1] | 88.2 (321/364) [84.6, 91.5] | 0.876 [0.827, 0.917] | 87.9 [84.1, 91.3] |
|  | Borderline | 61.5 (32/52) [47.8, 75.0] | 96.2 (300/312) [93.8, 98.1] |  |  |  |
|  | Malignant | 89.1 (115/129) [83.5, 94.3] | 94.5 (222/235) [91.4, 97.2] |  |  |  |
| The expert examiner | | | | | | |
| Reader 11 | Benign | 91.8 (168 183) [87.6, 95.5] | 89.5 (162 181) [84.8, 93.8] | 86.5 (315/364) [82.7, 89.8] | 0.843 [0.786, 0.892] | 86.2 [82.3, 89.7] |
|  | Borderline | 59.6 (31/52) [46.3, 72.4] | 96.2 (300/312) [93.9, 98.1] |  |  |  |
|  | Malignant | 89.9 (116/129) [84.4, 94.9] | 92.3 (217/235) [88.8, 95.5] |  |  |  |

Note. — Data in parentheses are numerators/denominators; data in brackets are 95% confidence intervals (CIs). 95% CIs were obtained by patient-level bootstrap (5,000 resamples). SPE = Specificity, SEN = Sensitivity, ACC = Accuracy, Clinical-OMTA = Clinical ovarian multi-task attention model.

Table S7. Radiologists’ subjective assessments of ultrasound images and clinical information in the external image test dataset.

| Readers | Classification | SEN (%) | SPE (%) | ACC (%) | Kappa | F1-score (%) |
| --- | --- | --- | --- | --- | --- | --- |
| Junior radiologists | | | | | | |
| Reader 1 | Benign | 48.2 (109/226) [41.7, 54.8] | 86.0. (98/114) [79.5, 91.9] | 52.6 (179/340) [47.4, 57.9] | 0.349 [0.265, 0.432] | 55.1 [49.7, 60.3] |
|  | Borderline | 18.9 (7/37) [6.8, 32.4] | 82.8 (251/303) [78.5, 87.1] |  |  |  |
|  | Malignant | 81.8 (63/77) [73.0, 90.1] | 64.6 (170/263) [47.4, 57.9] |  |  |  |
| Reader 2 | Benign | 82.7 (187/226) [77.7, 87.5] | 60.5 (69/114) [51.4, 69.4] | 72.1 (245/340) [67.4, 76.8] | 0.497 [0.398, 0.592] | 68.5 [63.1, 73.7] |
|  | Borderline | 0.0 (0/37) [0.0, 0.0] | 100 (303/303) [100, 100] |  |  |  |
|  | Malignant | 75.3 (58/77) [65.8, 84.6] | 81.0 (213/263) [76.2, 85.4] |  |  |  |
| Reader 3 | Benign | 81.9 (185/226) [76.7, 86.8] | 65.8 (75/114) [57.0, 74.5] | 71.2 (242/340) [66.2, 76.2] | 0.515 [0.418, 0.606] | 69.0 [63.8, 74.4] |
|  | Borderline | 2.7 (1/37) [0.0, 9.1] | 97.0 (294/303) [95.0, 98.7] |  |  |  |
|  | Malignant | 72.7 (56/77) [62.4, 82.5] | 81.0 (213/263) [76.2, 85.8] |  |  |  |
| Reader 4 | Benign | 96.5 (218/226) [93.9, 98.7] | 54.4 (62/114) [45.2, 63.7] | 80.0 (272/340) [75.6, 84.1] | 0.582 [0.478, 0.680] | 77.9 [72.7, 82.6] |
|  | Borderline | 32.4 (12/37) [17.9, 47.5] | 98.3 (298/303) [96.7, 99.7] |  |  |  |
|  | Malignant | 54.6 (42/77) [42.9, 65.5] | 95.8 (252/263) [93.3, 98.0] |  |  |  |
| Reader 5 | Benign | 93.8 (212/226) [90.4, 96.8] | 57.0 (65/114) [47.9, 65.7] | 78.2 (266/340) [73.8, 82.4] | 0.597 [0.493, 0.686] | 76.6 [71.6, 81.3] |
|  | Borderline | 29.7 (11/37) [15.4, 45.2] | 95.8 (289/303) [93.0, 97.7] |  |  |  |
|  | Malignant | 55.8 (43/77) [44.6, 66.7] | 95.8 (252/263) [93.4, 98.1] |  |  |  |
| Reader 6 | Benign | 40.3 (91/226) [33.8, 46.5] | 79.0 (90/114) [71.2, 86.2] | 43.5 (148/340) [38.2, 48.8] | 0.169 [0.086, 0.250] | 46.5 [41.1, 51.7] |
|  | Borderline | 21.6 (8/37) [9.1, 35.7] | 81.9 (248/303) [77.3, 86.1] |  |  |  |
|  | Malignant | 63.6 (49/77) [52.4, 74.3] | 57.0 (150/163) [51.1, 63.0] |  |  |  |
| Intermediate radiologists | | | | | | |
| Reader 7 | Benign | 85.4 (193/226) [80.6, 89.8] | 77.2 (88/114) [69.6, 85.0] | 74.7 (254/340) [70.0, 79.1] | 0.653 [0.565, 0.734] | 75.4 [70.6, 79.8] |
|  | Borderline | 29.7 (11/37) [15.2, 45.2] | 88.8 (269/303) [85.1, 92.1] |  |  |  |
|  | Malignant | 64.9 (50/77) [54.0, 75.4] | 90.1 (237/263) [86.4, 93.6] |  |  |  |
| Reader 8 | Benign | 70.4 (159/226) [64.3, 76.1] | 87.7 (100/114) [81.5, 93.6] | 69.7 (237/340) [64.7, 74.7] | 0.612 [0.530, 0.690] | 71.4 [66.5, 76.1] |
|  | Borderline | 27.0 (10/37) [13.2, 42.9] | 86.8 (263/303) [83.0, 90.4] |  |  |  |
|  | Malignant | 88.3 (68/77) [80.8, 95.1] | 81.4 (214/263) [76.6, 86.1] |  |  |  |
| Reader 9 | Benign | 98.7 (223/226) [96.9, 100] | 63.2 (72/114) [54.3, 72.0] | 80.6 (274/340) [76.5, 84.7] | 0.673 [0.583, 0.757] | 78.1 [73.1, 83.1] |
|  | Borderline | 21.6 (8/37) [8.9, 35.7] | 96.4 (292/303) [94.1, 98.4] |  |  |  |
|  | Malignant | 55.8 (43/77) [44.4, 67.1] | 95.1 (250/263) [92.3, 97.4] |  |  |  |
| Reader 10 | Benign | 93.8 (212/226) [90.4, 96.8] | 82.5 (94/114) [75.2, 89.3] | 85.3 (290/340) [81.5, 88.8] | 0.781 [0.705, 0.847] | 84.6 [80.3, 88.5] |
|  | Borderline | 40.5 (15/37) [24.4, 56.3] | 97.0 (294/303) [95.1, 98.7] |  |  |  |
|  | Malignant | 81.8 (63/77) [73.0, 90.1] | 92.0 (242/263) [88.6, 95.1] |  |  |  |
| The expert examiner | | | | | | |
| Reader 11 | Benign | 96.0 (217/226) [93.3, 98.3] | 81.6 (93/114) [74.0, 88.3] | 87.4 (297/340) [83.5, 90.6] | 0.821 [0.754, 0.878] | 86.2 [81.9, 90.0] |
|  | Borderline | 35.1 (13/37) [20.0, 51.3] | 98.7 (299/303) [97.3, 99.7] |  |  |  |
|  | Malignant | 87.0 (67/77) [79.2, 94.1] | 93.2 (245/263) [90.0, 96.1] |  |  |  |

Note. — Data in parentheses are numerators/denominators; data in brackets are 95% confidence intervals (CIs). 95% CIs were obtained by patient-level bootstrap (5,000 resamples). SPE = Specificity, SEN = Sensitivity, ACC = Accuracy.

Table S8. Clinical-OMTA-assisted evaluation of ultrasound images and clinical information in the external image test dataset.

| Readers | Classification | SEN (%) | SPE (%) | ACC (%) | Kappa | F1-score (%) |
| --- | --- | --- | --- | --- | --- | --- |
| Junior radiologists | | | | | | |
| Reader 1 | Benign | 94.2 (213/226) [91.1, 97.1] | 83.3 (95/114) [76.3, 89.9] | 85.0 (289/340) [81.2, 88.8] | 0.789 [0.718, 0.853] | 84.4 [80.2, 88.4] |
|  | Borderline | 40.5 (15/37) [25.0, 56.7] | 96.0 (291/303) [93.8, 98.1] |  |  |  |
|  | Malignant | 79.2 (61/77) [69.9, 88.1] | 92.4 (243/263) [89.1, 95.4] |  |  |  |
| Reader 2 | Benign | 94.2 (213/226) [91.1, 97.1] | 83.3 (95/114) [76.3, 89.9] | 85.9 (292/340) [82.1, 89.4] | 0.805 [0.737, 0.867] | 85.5 [81.4, 89.2] |
|  | Borderline | 45.9 (17/37) [30.0, 62.2] | 95.7 (290/303) [93.4, 98.0] |  |  |  |
|  | Malignant | 80.5 (62/77) [71.2, 89.2] | 93.9 (247/263) [90.9, 96.6] |  |  |  |
| Reader 3 | Benign | 94.7 (214/226) [91.7, 97.4] | 85.1 (97/114) [78.3, 91.3] | 86.5 (294/340) [82.6, 90.0] | 0.828 [0.765, 0.883] | 86.1 [82.1, 89.9] |
|  | Borderline | 45.9 (17/37) [30.0, 62.2] | 95.4 (289/303) [92.9, 97.6] |  |  |  |
|  | Malignant | 81.8 (63/77) [72.8, 90.3] | 94.3 (248/263) [91.3, 96.9] |  |  |  |
| Reader 4 | Benign | 97.3 (220/226) [95.1, 99.1] | 84.2 (96/114) [77.5, 90.6] | 87.9 (299/340) [84.4, 91.2] | 0.832 [0.769, 0.889] | 87.2 [83.3, 90.9] |
|  | Borderline | 45.9 (17/37) [30.0, 62.2] | 97.4 (295/303) [95.4, 99.0] |  |  |  |
|  | Malignant | 80.5 (62/77) [71.2, 89.2] | 94.3 (248/263) [91.3, 96.9] |  |  |  |
| Reader 5 | Benign | 96.5 (218/226) [93.9, 98.7] | 84.2 (96/114) [77.5, 90.4] | 87.6 (298/340) [84.1, 91.2] | 0.831 [0.767, 0.888] | 87.0 [83.1, 90.7] |
|  | Borderline | 45.9 (17/37) [30.0, 62.5] | 97.0 (294/303) [95.0, 98.7] |  |  |  |
|  | Malignant | 81.8 (63/77) [72.6, 90.2] | 94.3 (248/263) [91.4, 96.9] |  |  |  |
| Reader 6 | Benign | 94.7 (214/226) [91.7, 97.4] | 81.6 (93/114) [74.3, 88.3] | 85.6 (291/340) [81.8, 89.1] | 0.784 [0.711, 0.850] | 85.1 [81.0, 88.9] |
|  | Borderline | 45.9 (17/37) [30.0, 62.2] | 96.4 (292/303) [94.2, 98.3] |  |  |  |
|  | Malignant | 77.9 (60/77) [68.4, 0.87] | 93.5 (246/263) [90.5, 96.2] |  |  |  |
| Intermediate radiologists | | | | | | |
| Reader 7 | Benign | 96.5 (218/226) [93.8, 98.7] | 86.0 (98/114) [79.5, 92.0] | 87.4 (297/340) [83.8, 90.9] | 0.827 [0.763, 0.886] | 87.0 [83.2, 90.6] |
|  | Borderline | 51.4 (19/37) [35.3, 67.7] | 96.0 (291/303) [93.7, 98.0] |  |  |  |
|  | Malignant | 77.9 (60/77) [68.4, 86.9] | 94.3 (248/263) [91.4, 96.9] |  |  |  |
| Reader 8 | Benign | 96.9 (219/226) [94.4, 99.1] | 88.6 (101/114) [82.5, 94.0] | 90.9 (309/340) [87.6, 93.8] | 0.875 [0.819, 0.924] | 90.4 [86.7, 93.6] |
|  | Borderline | 54.1 (20/37) [37.8, 70.3] | 98.7 (299/303) [97.3, 99.7] |  |  |  |
|  | Malignant | 90.9 (70/77) [83.8, 96.9] | 94.7 (249/263) [91.9, 97.2] |  |  |  |
| Reader 9 | Benign | 99.1 (224/226) [97.7, 100] | 84.2 (96/114) [77.2, 90.7] | 89.7 (305/340) [86.5, 92.9] | 0.862 [0.804, 0.914] | 88.9 [85.3, 92.5] |
|  | Borderline | 48.6 (18/37) [32.6, 64.9] | 98.0 (297/303) [96.4, 99.4] |  |  |  |
|  | Malignant | 81.8 (63/77) [72.6, 90.2] | 958 (252/263) [93.3, 98.1] |  |  |  |
| Reader 10 | Benign | 99.1 (224/226) [97.7, 100] | 85.1 (97/114) [78.5, 91.4] | 91.5 (311/340) [88.5, 94.4] | 0.883 [0.829, 0.931] | 90.8 [87.2, 93.9] |
|  | Borderline | 51.4 (19/37) [35.1, 66.7] | 99.3 (301/303) [98.3, 100] |  |  |  |
|  | Malignant | 88.3 (68/77) [80.8, 95.1] | 96.2 (253/263) [93.8, 98.4] |  |  |  |
| The expert examiner | | | | | | |
| Reader 11 | Benign | 97.3 (220/226) [95.0, 99.1] | 86.8 (99/114) [80.3, 92.7] | 89.7 (305/340) [86.2, 92.6] | 0.879 [0.825 0.925] | 88.0 [ 85.0, 92.3] |
|  | Borderline | 43.2 (16/37) [27.6, 59.4] | 98.0 (297, 303) [96.3, 99.4] |  |  |  |
|  | Malignant | 89.6 (69/77) [82.3, 95.9] | 94.7 (249/263) [91.9, 97.3] |  |  |  |

Note. — Data in parentheses are numerators/denominators; data in brackets are 95% confidence intervals (CIs). 95% CIs were obtained by patient-level bootstrap (5,000 resamples). SPE = Specificity, SEN = Sensitivity, ACC = Accuracy, Clinical-OMTA = Clinical ovarian multi-task attention model.

Table S9. Reader-level change analysis in the internal test dataset.

| Readers | Corrected | Wrongly Reversed | Left  correct | Left  incorrect | Change-rate (%) | Net-benefit |
| --- | --- | --- | --- | --- | --- | --- |
| Junior radiologists  (average) | 78 | 14 | 224 | 48 | 25.3 | 64 |
| Reader 1 | 73 | 17 | 232 | 42 | 24.7 | 56 |
| Reader 2 | 65 | 22 | 226 | 51 | 23.9 | 43 |
| Reader 3 | 63 | 8 | 245 | 48 | 19.5 | 55 |
| Reader 4 | 90 | 10 | 217 | 47 | 27.5 | 80 |
| Reader 5 | 66 | 16 | 241 | 41 | 22.5 | 50 |
| Reader 6 | 113 | 11 | 180 | 60 | 34.0 | 102 |
| Intermediate radiologists  (average) | 55 | 9 | 259 | 41 | 17.6 | 46 |
| Reader 7 | 50 | 11 | 255 | 48 | 16.8 | 39 |
| Reader 8 | 68 | 5 | 245 | 46 | 20.1 | 63 |
| Reader 9 | 69 | 19 | 249 | 27 | 24.2 | 50 |
| Reader 10 | 33 | 2 | 288 | 41 | 9.6 | 31 |
| The expert examiner |  |  |  |  |  |  |
| Reader 11 | 27 | 9 | 288 | 40 | 9.9 | 18 |

Note. — For each of the 11 radiologists, we reported (i) Corrected: cases where the AI converted an incorrect decision to correct, (ii) Wrongly Reversed: cases where it converted a correct decision to incorrect, (iii) Left correct: cases that remained correct, and (iv) Left incorrect: cases that remained incorrect. Change rate = (i + ii) / (i + ii + iii + iv) and net benefit = (i − ii). Group averages for the four metrics (Corrected, Wrongly reversed, Left correct, and Left incorrect) were calculated as the arithmetic mean of the individual reader values within the group (rounded to the nearest integer). These four group-averaged counts were then used to calculate the group-level change rate and net benefit.

Table S10. Reader-level change analysis in the external image test dataset.

| Readers | Corrected | Wrongly reversed | Left  correct | Left incorrect | Change-rate (%) | Net-benefit |
| --- | --- | --- | --- | --- | --- | --- |
| Junior radiologists  (average) | 79 | 11 | 215 | 35 | 26.5 | 68 |
| Reader 1 | 120 | 10 | 169 | 41 | 38.2 | 110 |
| Reader 2 | 63 | 16 | 229 | 32 | 23.2 | 47 |
| Reader 3 | 62 | 10 | 232 | 36 | 21.2 | 52 |
| Reader 4 | 37 | 10 | 262 | 31 | 13.8 | 27 |
| Reader 5 | 41 | 9 | 257 | 33 | 14.7 | 32 |
| Reader 6 | 153 | 10 | 138 | 39 | 47.9 | 143 |
| Intermediate radiologists  (average) | 46 | 6 | 258 | 31 | 15.3 | 40 |
| Reader 7 | 53 | 10 | 244 | 33 | 18.5 | 43 |
| Reader 8 | 73 | 1 | 236 | 30 | 21.8 | 72 |
| Reader 9 | 33 | 2 | 272 | 33 | 10.3 | 31 |
| Reader 10 | 24 | 9 | 281 | 26 | 9.7 | 15 |
| The expert examiner |  |  |  |  |  |  |
| Reader 11 | 16 | 2 | 295 | 27 | 5.3 | 14 |

Note. — For each of the 11 radiologists, we reported (i) Corrected: cases where the AI converted an incorrect decision to correct, (ii) Wrongly reversed: cases where it converted a correct decision to incorrect, (iii) Left correct: cases that remained correct, and (iv) Left incorrect: cases that remained incorrect. Change rate = (i + ii) / (i + ii + iii + iv) and net benefit = (i − ii). Group averages for the four metrics (Corrected, Wrongly reversed, Left correct, and Left incorrect) were calculated as the arithmetic mean of the individual reader values within the group (rounded to the nearest integer). These four group-averaged counts were then used to calculate the group-level change rate and net benefit.

Table S11. The comparison of performance of three methods in the external video test dataset: the fully-automated video pipeline, a pipeline in which an expert examiner manually corrected the ROI bounding box of each frame after U-Net extraction, and a method based on three representative frames.

| Method | Accuracy (%) | Benign AUC | Borderline AUC | Malignant AUC |
| --- | --- | --- | --- | --- |
| Fully-automated video pipeline * | 79.3 (126/159) [72.9, 84.9] | 0.908 [0.894, 0.927] | 0.907 [0.887, 0.925] | 0. 952 [0.940, 0.966] |
| Expert manually correcting | 79.9 (127/159) [73.6, 86.2] | 0.907 [0.888, 0.927] | 0.903 [0.879, 0.926] | 0.952 [0.939, 0.968] |
| Three representative frames | 78.6 (125/159) [72.3, 84.9] | 0.932 [0.916, 0.946] | 0.901 [0.878, 0.935] | 0.962 [0.952, 0.973] |

Note. — Data in parentheses are numerators/denominators. Data in brackets are 95% confidence intervals (CIs). 95% CIs were obtained by patient-level bootstrap (5,000 resamples). Three methods in the external video test dataset were based on Clinical-OMTA. * For the fully-automated video pipeline, when multiple ROIs were detected in a single frame, each ROI was independently classified by the Clinical-OMTA model. The prediction probability from the ROI with the largest area was then selected as the representative value for that frame. AUC = Area under the receiver operating characteristic curve, ROI = Region of interest, Clinical-OMTA = Clinical ovarian multi-task attention model.

Table S12. Vendor distribution and scanning methods in two hospitals of external image test dataset.

|  |  | Hospital A in northern centre (n = 237) | Hospital B in southern centre (n =103) | |
| --- | --- | --- | --- | --- |
| Vendors |  |  |  |  |
| GE | LOGIQ E9 | 2 (0.8) | — |  |
|  | Voluson E10 | 15 (6.3) | — |  |
|  | Voluson E8 | 142 (59.9) | 55 (53.4) |  |
| Philips | Affiniti70 | 45 (19.0) | — |  |
|  | HD15 | 12 (5.1) | — |  |
| Mindray | Resona 8 | 7 (2.9) | 27 (26.2) |  |
|  | Resona 7 | — | 2 (1.9) |  |
|  | Resona 8S | — | 2 (1.9) |  |
|  | Resona R7S | — | 1 (1.0) |  |
| HITACHI | Preirus | 11 (4.6) | — |  |
| Samsung | MEDISON-4 | 2 (0.8) | — |  |
|  | medisionV20-2 | — | 1 (1.0) |  |
|  | W102 | — | 1 (1.0) |  |
|  | W10A | — | 3 (2.9) |  |
|  | WS80A | 1 (0.4) | 2 (1.9) |  |
| SonoScape | S60 | — | 8 (7.8) |  |
| SuperSonic Imagine | AIXplorer | — | 1 (1.0) |  |
| Scanning methods |  |  |  |  |
| Transvaginal |  | 180 (75.9) | 82 (79.6) |  |
| Transabdominal |  | 57 (24.1) | 21 (20.4) |  |

Note. — Data are numbers of patients with percentages in parentheses. Hospital A in northern centre = Second Affiliated Hospital of Harbin Medical University in Heilongjiang Province, Hospital B in southern centre = Sichuan Provincial Maternity and Child Health Care Hospital in Sichuan Province.

Table S13. Generalisation of Clinical-OMTA based on different histologic subtypes in the external image test dataset.

| Histologic subtypes | Accuracy (%) | Benign AUC | Borderline AUC | Malignant AUC |
| --- | --- | --- | --- | --- |
| Germ cell | 94.5 (138/146)  [90.8, 98.2] | 0.959 [0.871, 1.000] | 0.924 [0.876, 0.965] | 0.940 [0.830, 1.000] |
| Serous | 83.8 (57/68)  [75.1, 92.6] | 0.928 [0.855, 0.983] | 0.774 [0.567, 0.948] | 0.900 [0.816, 0.970] |
| Mucinous | 69.4 (25/36)  [54.4, 84.5] | 0.899 [0.773, 0.990] | 0.799 [0.641, 0.928] | 0.889 [0.735, 1.000] |
| Sex cord stromal | 75.0 (15/20)  [56.0, 94.0] | 0.879 [0.688, 1.000] | 0.632 [0.421, 0.842] | 0.893 [0.714, 1.000] |
| Mixed * | 75.0 (15/20)  [56.0, 94.0] | 0.949 [0.824, 1.000] | 0.880 [0.667, 1.000] | 0.844 [0.569, 1.000] |
| Others ** | 82.0 (41/50)  [71.4, 92.7] | 0.939 [0.868, 0.993] | —— | 0.932 [0.851, 0.990] |

**Note.** — Data in parentheses are numerators/denominators. Data in brackets are 95% confidence intervals (CIs). 95% CIs were obtained by patient-level bootstrap (5,000 resamples). * Mixed histology of adnexal mass indicates the mass contains multiple pathological subtypes simultaneously. ** Other histology encompassed clear cell endometrioid, metastatic adnexal tumour, mesenchymal tumour, Brenner tumour, etc. AUC = Area under the receiver operating characteristic curve. AUC were based on 340 patient-level predictions in the external image test dataset. Clinical-OMTA = Clinical ovarian multi-task attention model.

Table S14. Comparison of diagnostic performance for adnexal tumour classification using different feature fusion approaches in MTANet architectures with and without segmentation branch in the external image test dataset.

| Segmentation branch | Backbone variant | Classifier training | Feature fusion | | | | | Accuracy  (%) | F1-score  (%) | Benign  AUC | Borderline  AUC | Malignant  AUC |  |
| --- | --- | --- | --- | --- | --- | --- | --- | --- | --- | --- | --- | --- | --- |
|  |  |  | $\boldsymbol{f}_{\boldsymbol{Benign}}$ | $\boldsymbol{f}_{\boldsymbol{Malignant}}$ | Probabilities (three dimensions) | CA125 | Age |  |  |  |  |  |  |
| Yes | MTANet_BvNB + MTANet_MvB | FC Layer* |  |  | √ | √ | √ | 85.6 (291/340)  [81.8, 89.1] | 85.4  [81.4, 89.1] | 0.950  [0.938, 0.962] | 0.870  [0.842, 0.902] | 0.930  [0.913, 0.948] |  |
| Yes | | MTANet_BvNB + MTANet_MvB | end-to-end** |  |  | √ |  |  | 83.5 (284/340)  [79.4, 87.4] | 83.6  [79.5, 87.4] | 0.943  [0.929, 0.963] | 0.834  [0.796, 0.875] | 0.919  [0.898, 0.944] |
| Yes | MTANet_BvNB + MTANet_MvB | FC Layer *** |  | √ | √ | √ | √ | 85.3 (290/340)  [81.5, 88.8] | 85.1 [81.1, 88.8] | 0.946  [0.930, 0.966] | 0.846  [0.805, 0.898] | 0.930 [0.912, 0.949] |  |
| Yes | MTANet_BvNB + MTANet_MvB | FC Layer **** |  | √ | √ |  |  | 83.8 (285/340)  [79.7, 87.7] | 83.9 [79.8, 87.7] | 0.938 [0.919, 0.960] | 0.836  [0.798, 0.893] | 0.917 [0.895, 0.939] |  |
| No | MTANet_BvNB + MTANet_MvB | FC Layer |  | √ | √ | √ | √ | 72.4 (246/340) [67.7, 77.1] | 69.5 [64.0, 75.1] | 0.770 [0.741, 0.806] | 0.606  [0.534, 0.688] | 0.853 [0.815, 0.883] |  |
| No | MTANet_BvNB + MTANet_MvB | FC Layer | √ | √ | √ | √ | √ | 67.7 (230/340)  [62.7, 72.7] | 69.2 [64.4, 74.0] | 0.777  [0.749, 0.801] | 0.659  [0.607, 0.731] | 0.841 [0.815, 0.867] |  |
| No | MTANet_BvNB + MTANet_MvB | FC Layer |  | √ | √ |  |  | 68.8 (234/340) [63.8, 73.8] | 67.9 [62.8, 73.2] | 0.739 [0.705, 0.767] | 0.612  [0.550, 0.691] | 0.802 [0.771, 0.833] |  |
| No | MTANet_BvNB + MTANet_MvB | FC Layer | √ |  | √ | √ | √ | 66.8 (227/340)  [61.5, 71.8] | 69.1 [64.2, 73.7] | 0.799 [0.772, 0.824] | 0.689  [0.644, 0.758] | 0.854 [0.832, 0.879] |  |
| No | MTANet_BvNB + MTANet_MvB | end-to-end |  |  | √ |  |  | 72.4  (246/340) [67.4, 77.1] | 73.2  [68.4, 77.8] | 0.813 [0.787, 0.839] | 0.715  [0.664, 0.774] | 0.846 [0.823, 0.869] |  |
| Segmentation branch | Backbone variant | Classifier training | $\boldsymbol{f}$ | | Probabilities (three dimensions”) | CA125 | Age | Accuracy  (%) | F1-score  (%) | Benign  AUC | Borderline  AUC | Malignant  AUC |  |
| No | MTANet_BBM | FC Layer argmax | √ | | √ | √ | √ | 75.9 (258/340)  [71.2, 80.3] | 76.0 [71.3, 80.7] | 0.823 [0.794, 0.849] | 0.719  [0.670, 0.787] | 0.901 [0.879, 0.918] |  |
| No | MTANet_BBM | end-to-end argmax |  | | √ |  |  | 75.6 (257/340) [70.9, 80.0] | 75.5 [70.8, 80.2] | 0.842 [0.814, 0.867] | 0.759  [0.715, 0.815] | 0.895 [0.869, 0.914] |  |
| Yes | MTANet_BBM | end-to-end argmax |  | | √ |  |  | 73.5  (250/340)  [68.8, 78.2] | 69.2 [63.4, 74.8] | 0.871  [0.853, 0.898] | 0.735  [0.691, 0.789] | 0.887  [0.868, 0.909] |  |

Note. — Data in parentheses are numerators/denominators. Data in brackets are 95% confidence intervals (CIs). 95% CIs were obtained by patient-level bootstrap (5,000 resamples). The study employed three backbone variants: MTANet_BBM (Benign, Borderline, and Malignant) for differentiating benign, borderline, and malignant adnexal masses; MTANet_BvNB (Benign vs. Non-Benign) for differentiating between benign and non-benign adnexal masses; and MTANet_MvB (Malignant vs. Borderline) for differentiating malignant from borderline adnexal masses. *End-to-end* refers to a fully-connected classifier trained jointly with the backbone and did not involve feature fusion. *Argmax* refers to selecting the class with the highest probability among the three as the predicted category instead of using two thresholds.$\boldsymbol{f}_{\boldsymbol{Benign}}$ refers to the feature vector taken just before the final FC layer of MTANet_BvNB, while$\boldsymbol{f}_{\boldsymbol{Malignant}}$ refers to the feature vector taken just before the final FC layer of MTANet_MvB. Similarly, $\boldsymbol{f}$ represents the feature vector before the last FC layer of MTANet_BBM. Except for the features of the end-to-end model that only involve three-dimensional output features, models using FC layer classification heads use checkmarks √ to represent the features involved in the training of the classification head. The features that were checked (√) were concatenated and used as input features for the classification head. The age and CA125 characteristics were standardised using the same method as in the main text. * The first row is the same as “Clinical-OMTA” in the main results. ** The second row was the same as “OMTA” in the main results. *** The model in the third row fused the probabilities $\boldsymbol{f}_{\boldsymbol{Malignant}}$, $\boldsymbol{p}_{\mathbf{Benign}}$, ${\hat{\boldsymbol{p}}}_{\mathbf{Borderline}}$, ${\hat{\boldsymbol{p}}}_{\mathbf{Malignant}}$, age, and CA125. Then output the predicted probability as result. **** The model in the fourth row fused the probabilities $\boldsymbol{f}_{\boldsymbol{Malignant}}$, $\boldsymbol{p}_{\mathbf{Benign}}$, ${\hat{\boldsymbol{p}}}_{\mathbf{Borderline}}$, and ${\hat{\boldsymbol{p}}}_{\mathbf{Malignant}}$. Then output the predicted probability as result. AUC = Area under the receiver operating characteristic curve, FC = Fully connected, CA125 = Carbohydrate antigen 125, MTANet = Multi-task attention network.

Table S15-1. Performance comparison of different imputation methods with FC in the external image test dataset.

| Classification head | Imputation method | Separate “missing” indicator | Accuracy (%) | Benign AUC | Borderline AUC | Malignant AUC |
| --- | --- | --- | --- | --- | --- | --- |
| FC | -1 |  | 86.2 (293/340) [82.4 89.7] | 0.943 [0.926, 0.957] | 0.841 [0.797, 0.877] | 0.922 [0.898, 0.943] |
| FC | Mean |  | 85.0 (289/340) [81.2, 88.8] | 0.938 [0.916, 0.953] | 0.830 [0.791, 0.867] | 0.924 [0.901, 0.946] |
| FC | Median |  | 85.3 (290/340) [81.5, 88.8] | 0.944 [0.928, 0.958] | 0.846 [0.803, 0.883] | 0.925 [0.903, 0.946] |
| FC | KNN |  | 85.0 (289/340) [81.2, 88.8] | 0.946 [0.931, 0.961] | 0.848 [0.805, 0.884] | 0.926 [0.904, 0.947] |
| FC | MICE |  | 84.7 (288/340) [80.9, 88.5] | 0.946 [0.930, 0.960] | 0.847 [0.805, 0.884] | 0.927 [0.906, 0.947] |
| FC | -1 | √ | 85.0 (289/340) [81.2 88.8] | 0.943 [0.925, 0.957] | 0.842 [0.800, 0.880] | 0.925 [0.902, 0.945] |
| FC | Mean | √ | 85.0 (289/340) [81.2, 88.5] | 0.938 [0.916, 0.953] | 0.830 [0.791, 0.867] | 0.924 [0.901, 0.946] |
| FC | Median | √ | 84.7 (288/340) [80.9 88.5] | 0.935 [0.913, 0.951] | 0.825 [0.787, 0.863] | 0.924 [0.901, 0.945] |
| FC | KNN | √ | 84.7 (288/340) [80.9, 88.2] | 0.936 [0.914, 0.953] | 0.824 [0.785, 0.863] | 0.924 [0.901, 0.946] |
| FC | MICE | √ | 85.0 (289/340) [81.2 88.5] | 0.936 [0.914, 0.953] | 0.822 [0.782, 0.862] | 0.927 [0.904, 0.947] |

Note. — Data in parentheses are numerators/denominators. Data in brackets are 95% confidence intervals (CIs). 95% CIs were obtained by patient-level bootstrap (5,000 resamples). FC = Fully connected, AUC = Area under the receiver operating characteristic curve, KNN = K-nearest neighbors, MICE = Multiple imputation by chained equations. Table S15-2. Performance comparison of different imputation methods with other machine learning models in the external image test dataset.

| Classification  head | Imputation method | Separate “missing” indicator | Hyper-parameters | Accuracy (%) | Benign AUC | Borderline AUC | Malignant AUC |
| --- | --- | --- | --- | --- | --- | --- | --- |
| LR (TPOT) | Mean |  | C = 15, solver = newton-cg | 84.1 (286/340)  [80.0, 87.9] | 0.948  [0.933, 0.959] | 0.848  [0.809, 0.897] | 0.929  [0.910, 0.950] |
| LR (TPOT) | Median |  | C = 0.4, solver = newton-cg | 84.7 (288/340)  [80.8, 88.5] | 0.952  [0.935, 0.965] | 0.840  [0.803, 0.890] | 0.925  [0.904, 0.948] |
| LR (TPOT) | KNN |  | C = 1, solver = liblinear | 86.2 (293/340)  [82.4, 89.7] | 0.951  [0.938, 0.964] | 0.837  [0.795, 0.887] | 0.928  [0.909, 0.950] |
| LR (TPOT) | MICE |  | C = 5, solver = newton-cg | 85.6 (291/340)  [81.8, 89.1] | 0.952  [0.938, 0.963] | 0.843  [0.802, 0.886] | 0.926  [0.904, 0.951] |
| LR (TPOT) | Mean | √ | C = 15, solver = liblinear | 84.7 (292/340)  [80.9, 88.2] | 0.943  [0.924, 0.958] | 0.816  [0.776, 0.870] | 0.928  [0.908, 0.949] |
| LR (TPOT) | Median | √ | C = 5, solver = newton-cg | 84.4 (287/340)  [80.6, 88.2] | 0.941  [0.923, 0.957] | 0.815  [0.774, 0.865] | 0.922  [0.902, 0.944] |
| LR (TPOT) | KNN | √ | C = 10, solver = liblinear | 84.1 (286/340)  [80.0, 87.9] | 0.951  [0.938, 0.964] | 0.837  [0.795, 0.887] | 0.928  [0.909, 0.950] |
| LR (TPOT) | MICE | √ | C = 15, solver = liblinear | 84.4 (287/340)  [80.6, 88.2] | 0.941  [0.923, 0.957] | 0.814  [0.773, 0.862] | 0.930  [0.912, 0.950] |
| RF (TPOT) | Median |  | criterion = entropy, max_depth = 60, max_features = sqrt, min_samples_leaf = 5, min_samples_split = 10, n_estimators = 16 | 82.9 (282/340) [79.1, 86.8] | 0.942  [0.926, 0.959] | 0.803  [0.759, 0.848] | 0.919  [0.899, 0.942] |
| RF (TPOT) | Mean |  | criterion = entropy, max_depth = 30, max_features = sqrt, min_samples_leaf = 5, min_samples_split = 15, n_estimators = 16 | 82.1 (279/340) [77.9, 86.2] | 0.933  [0.914, 0.952] | 0.804  [0.765, 0.856] | 0.911  [0.891, 0.934] |
| RF (TPOT) | KNN |  | criterion = gini,  max_depth = 30, max_features = sqrt, min_samples_leaf = 5, min_samples_split = 2, n_estimators = 14 | 81.8 (278/340) [77.7, 85.6] | 0.944  [0.927, 0.959] | 0.793  [0.754, 0.858] | 0.919  [0.892, 0.947] |
| RF (TPOT) | MICE |  | criterion = gini,  max_depth = 20, max_features = sqrt, min_samples_leaf = 5, min_samples_split = 2, n_estimators = 14 | 81.8 (278/340) [77.7, 85.9] | 0.942  [0.927, 0.963] | 0.796  [0.751, 0.845] | 0.917  [0.898, 0.943] |
| RF (TPOT) | Median | √ | criterion = entropy, max_depth = 30, max_features = sqrt, min_samples_leaf = 5, min_samples_split = 15, n_estimators = 15 | 81.8 (278/340) [77.7, 85.6] | 0.940  [0.927, 0.958] | 0.799  [0.757, 0.844] | 0.918  [0.900, 0.940] |
| RF (TPOT) | Mean | √ | criterion = gini,  max_depth = 20, max_features = sqrt, min_samples_leaf = 10, min_samples_split =2, n_estimators = 14 | 82.7 (281/340) [78.5, 86.4] | 0.938  [0.922, 0.954] | 0.796  [0.767, 0.846] | 0.914  [0.885, 0.943] |
| RF (TPOT) | KNN | √ | criterion = gini,  max_depth = 60, max_features = sqrt, min_samples_leaf = 1, min_samples_split = 10, n_estimators = 13 | 79.7 (271/340) [75.3, 83.8] | 0.932  [0.913, 0.953] | 0.819  [0.782, 0.867] | 0.903  [0.872, 0.934] |
| RF (TPOT) | MICE | √ | criterion = gini,  max_depth = 60, max_features = sqrt, min_samples_leaf = 1, min_samples_split = 10, n_estimators = 13 | 81.8 (278/340) [77.4, 85.9] | 0.944  [0.928, 0.962] | 0.815  [0.770, 0.861] | 0.913  [0.893, 0.941] |
| SVC (TPOT) | Median |  | C = 5, decision_function_shape = ovo, gamma = auto, kernel = rbf | 85.0 (289/340) [81.2, 88.8] | 0.950  [0.932, 0.966] | 0.853  [0.819, 0.894] | 0.923  [0.908, 0.945] |
| SVC (TPOT) | Mean |  | C = 1, decision_function_shape = ovr, gamma = auto, kernel = linear | 84.7 (288/340) [80.9, 88.5] | 0.948  [0.932, 0.965] | 0.862  [0.826, 0.897] | 0.925  [0.910, 0.944] |
| SVC (TPOT) | KNN |  | C = 0.5, decision_function_shape = ovr, gamma = auto, kernel = linear | 85.3 (290/340) [81.5, 89.1] | 0.950  [0.937, 0.962] | 0.862  [0.835, 0.907] | 0.927  [0.909, 0.945] |
| SVC (TPOT) | MICE |  | C = 0.4, decision_function_shape = ovr, gamma = auto, kernel = linear | 85.3 (290/340) [81.5, 89.1] | 0.951  [0.938, 0.962] | 0.858  [0.826, 0.906] | 0.928  [0.908, 0.945] |
| SVC (TPOT) | Median | √ | C = 0.3, decision_function_shape = ovr, gamma = auto, kernel = poly | 85.3 (290/340) [81.5, 88.8] | 0.948  [0.930, 0.961] | 0.849  [0.814, 0.898] | 0.929  [0.910, 0.945] |
| SVC (TPOT) | Mean | √ | C = 15, decision_function_shape = ovr, gamma=auto, kernel = rbf | 84.7 (288/340) [80.9, 88.5] | 0.946  [0.929, 0.958] | 0.846  [0.811, 0.897] | 0.913  [0.887, 0.936] |
| SVC (TPOT) | KNN | √ | C = 15, decision_function_shape = ovo, gamma = auto, kernel = rbf | 84.7 (288/340) [80.9, 88.5] | 0.947  [0.928, 0.958] | 0.839  [0.804, 0.893] | 0.914  [0.888, 0.937] |
| SVC (TPOT) | MICE | √ | C = 0.1, decision_function_shape = ovo, gamma = auto, kernel = linear | 84.7 (288/340)  [80.9, 88.2] | 0.946  [0.929, 0.964] | 0.835  [0.796, 0.889] | 0.914  [0.888, 0.936] |
| SVC (default) | Median |  |  | 85.3 (290/340)  [81.5, 89.1] | 0.948  [0.932, 0.960] | 0.853  [0.818, 0.887] | 0.919  [0.891, 0.945] |
| SVC (default) | Mean |  |  | 85.3 (290/340)  [81.5, 89.1] | 0.948  [0.932, 0.961] | 0.855  [0.818, 0.888] | 0.920  [0.893, 0.946] |
| SVC (default) | KNN |  |  | 85.3 (290/340)  [81.5, 89.1] | 0.945  [0.929, 0.958] | 0.849  [0.815, 0.882] | 0.918  [0.892, 0.945] |
| SVC (default) | MICE |  |  | 85.3 (290/340)  [81.5, 89.2] | 0.949  [0.933, 0.961] | 0.856  [0.819, 0.888] | 0.921  [0.894, 0.946] |
| SVC (default) | Median | √ |  | 85.0 (289/340)  [81.2, 88.8] | 0.945  [0.929, 0.958] | 0.849  [0.816, 0.883] | 0.918  [0.891, 0.945] |
| SVC (default) | Mean | √ |  | 85.3 (290/340)  [81.5, 89.1] | 0.946  [0.930, 0.959] | 0.850  [0.816, 0.884] | 0.919  [0.892, 0.945] |
| SVC (TPOT) | KNN | √ |  | 85.3 (290/340)  [81.5, 89.1] | 0.948  [0.933, 0.961] | 0.855  [0.819, 0.888] | 0.919  [0.892, 0.946] |
| SVC (TPOT) | MICE | √ |  | 85.3 (290/340)  [81.5, 88.8] | 0.944  [0.927, 0.957] | 0.850  [0.816, 0.883] | 0.921  [0.895, 0.946] |

Note. — Data in parentheses are numerators/denominators. Data in brackets are 95% confidence intervals (CIs). 95% CIs were obtained by patient-level bootstrap (5,000 resamples). RF candidate parameters: n_estimators = [10, 11, 12, 13, 14, 15, 16]; max_depth = [5, 10, 20, 30, 40, 50, 60]; criterion = [‘entropy’, ‘gini’]; min_samples_leaf = [1, 2, 5, 10]; min_samples_split = [2, 5, 10, 15]; max_features = [‘auto’, ‘sqrt’, ‘log2’]; LR candidate parameters: Cs = [0.1, 0.3, 0.4, 0.5, 0.8, 1, 5, 10, 15]; solvers = [‘liblinear’, ‘newton-cg’]; SVC candidate parameters: Cs = [0.3, 0.4, 0.5, 0.8, 1, 5, 10, 15]; kernels = [‘rbf’, ‘linear’, ‘poly’, ‘sigmoid’]; decision_function_shape = [‘ovo’, ‘ovr’]. Using TPOT for parameter selection, with the following TPOT parameter settings: (generations = 5, population_size = 24, offspring_size = 12, early_stop = 12, scoring = ‘accuracy’). SVC = Support vector classifier, AUC = Area under the receiver operating characteristic curve, KNN = K-nearest neighbors, MICE = Multiple imputation by chained equations, LR = Logistic regression, RF = Random forest. TPOT = Tree-based pipeline optimisation tool.

Figures


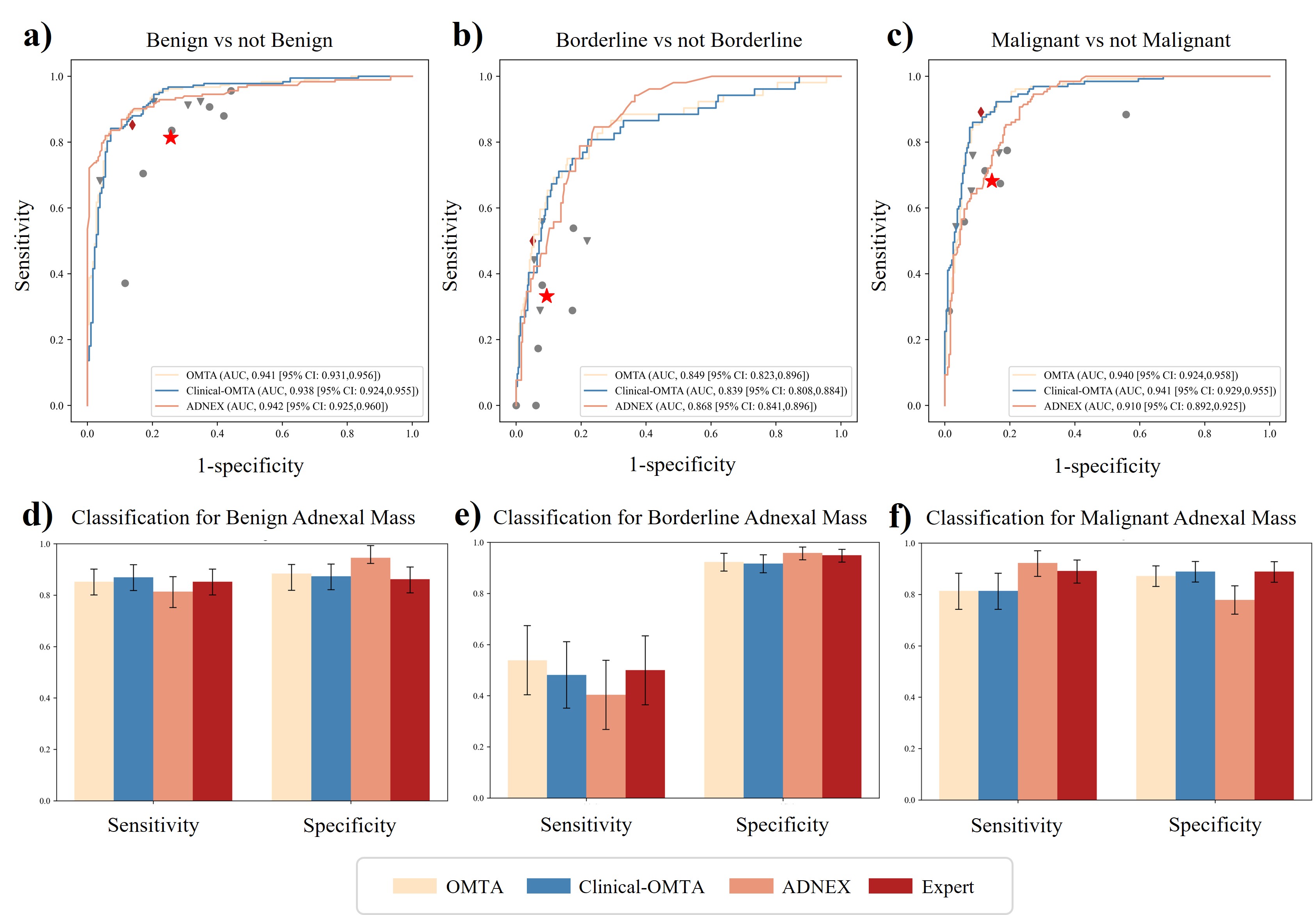


Figure S1. Diagnostic performance of OMTA, Clinical-OMTA, ADNEX, subjective assessment by an expert examiner, and radiologists with varying levels of experience in distinguishing benign, borderline, and malignant adnexal tumours in the internal test dataset. (a-c) show ROC curves of OMTA, Clinical-OMTA, and ADNEX. The red star represents the average performance of the 11 radiologists, where sensitivities and specificities were averaged across radiologists. Grey circles represent the performance of junior radiologists, grey triangles represent intermediate radiologists, and red diamonds represent subjective assessment by an expert examiner. ROC curves were based on 364 patient-level predictions in the internal test datasets. (d-f) show bar charts of sensitivity and specificity. 95% CIs were obtained by patient-level bootstrap (5,000 resamples). OMTA = Ovarian multi-task attention model, Clinical-OMTA = Clinical ovarian multi-task attention model, ADNEX = Assessment of Different NEoplasias in the adneXa, AUC = Area under the receiver operating characteristic curve, ROC = Receiver operating characteristic.


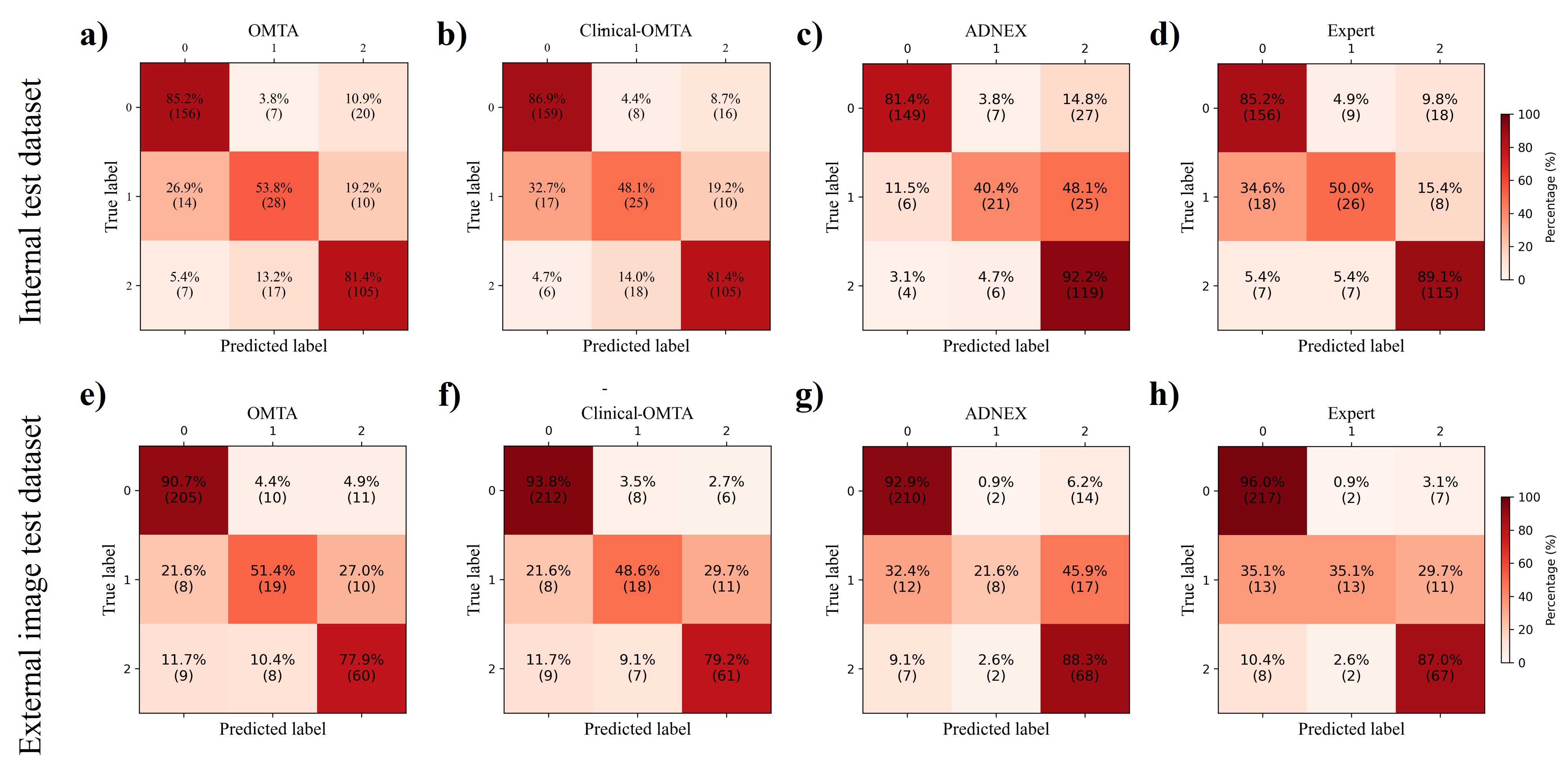


Figure S2. Confusion matrices demonstrating the diagnostic performance of different models and subjective assessment by an expert examiner in discriminating benign, borderline, and malignant adnexal tumours in the internal and external image test datasets. (a-d) Internal test dataset results for Clinical-OMTA (a), OMTA (b), ADNEX (c), and Expert (d); (e-h) External image test dataset results for Clinical-OMTA (e), OMTA (f), ADNEX (g), and Expert (h). Confusion matrices were based on 364 and 340 patient-level predictions in the internal and external test datasets, respectively. OMTA = Ovarian multi-task attention model, Clinical-OMTA = Clinical ovarian multi-task attention model, ADNEX = Assessment of Different NEoplasias in the adneXa.


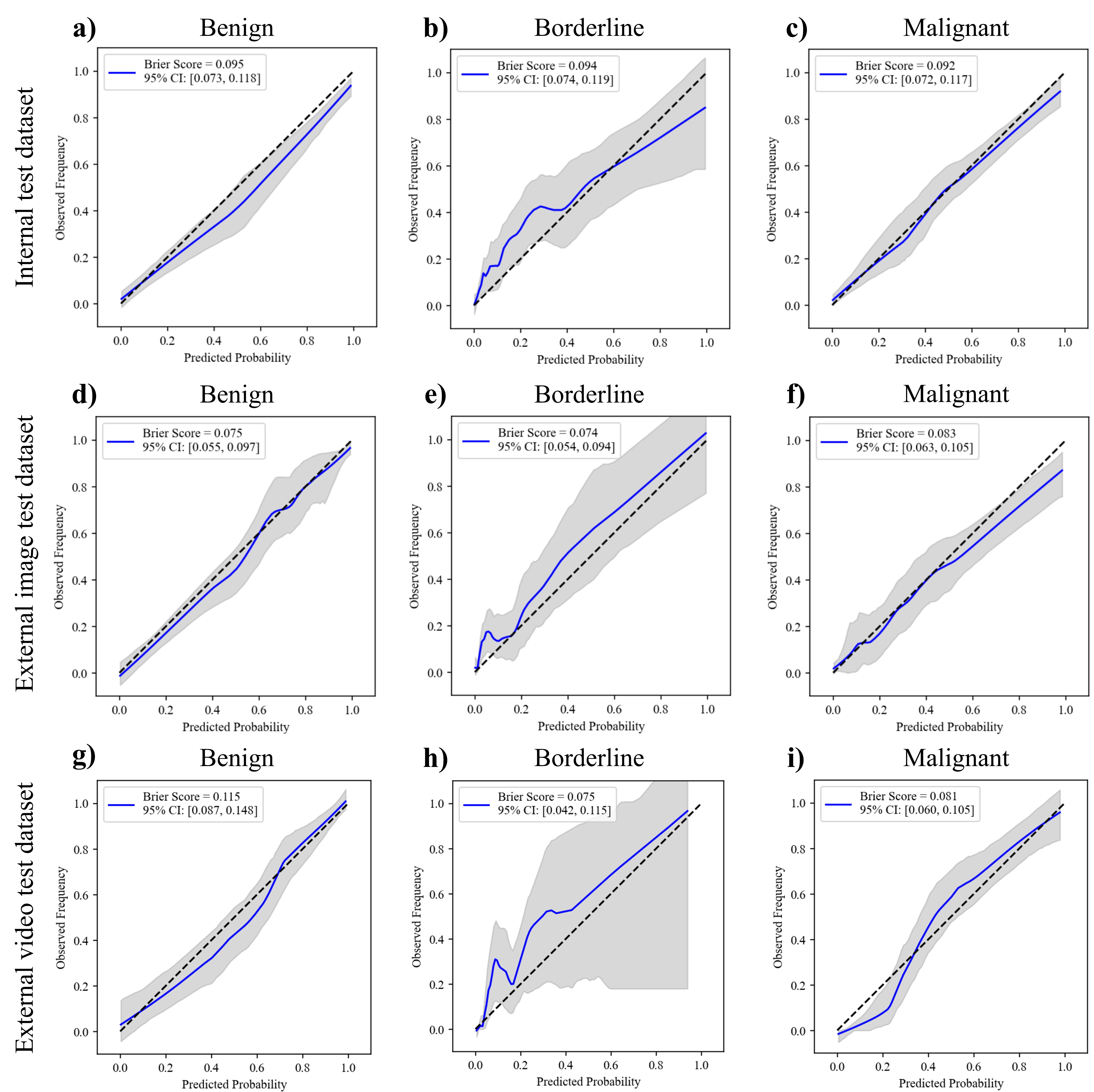


Figure S3. Calibration curves of benign, borderline, and malignant adnexal tumours in internal test dataset (a-c), external image test dataset (d-f), and video test dataset with expert manually correcting (g-i). The dashed line is the ideal calibration curve, where the predicted risks precisely match the observed outcomes. The fitted linear logistic calibration curves are shown in blue with 95% confidence bands in grey, depicting the relationship between the predicted risk of benign, borderline, and malignant adnexal masses and the actual observed proportion of benign, borderline, and malignant adnexal masses. The 95% confidence interval was calculated through 5,000 resampling operations using the bootstrap method. Calibration curves were based on 364, 340, and 159 patient-level predictions in the internal test dataset, external image test dataset, and video test dataset, respectively. The calibration curves evaluate the predictive performance of the Clinical-OMTA model. Clinical-OMTA = Clinical ovarian multi-task attention model.


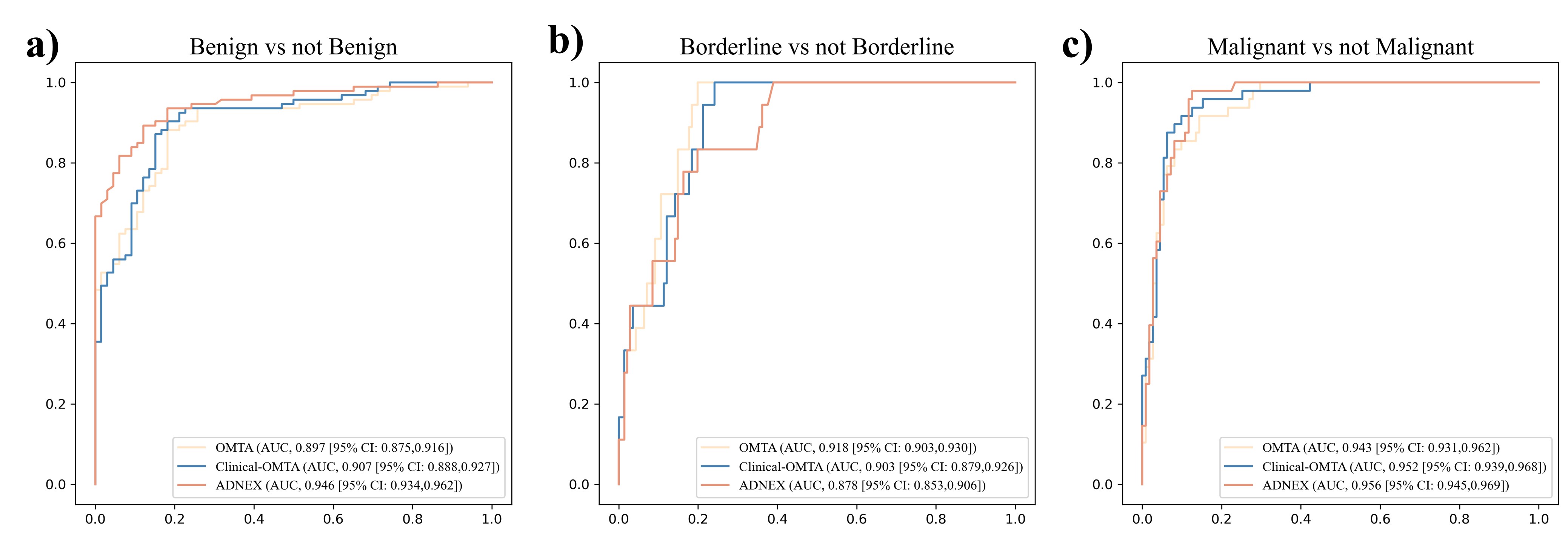
Figure S4. Receiver operating characteristic curves of external video test dataset with expert manually correcting showed the diagnostic performance of OMTA, Clinical-OMTA, and ADNEX to discriminate benign (a), borderline (b), and malignant (c) adnexal tumours. 95% CIs were obtained by patient-level bootstrap (5,000 resamples). The curves were based on 159 patient-level predictions. OMTA = Ovarian multi-task attention model, Clinical-OMTA = Clinical ovarian multi-task attention model, ADNEX = Assessment of Different NEoplasias in the adneXa.


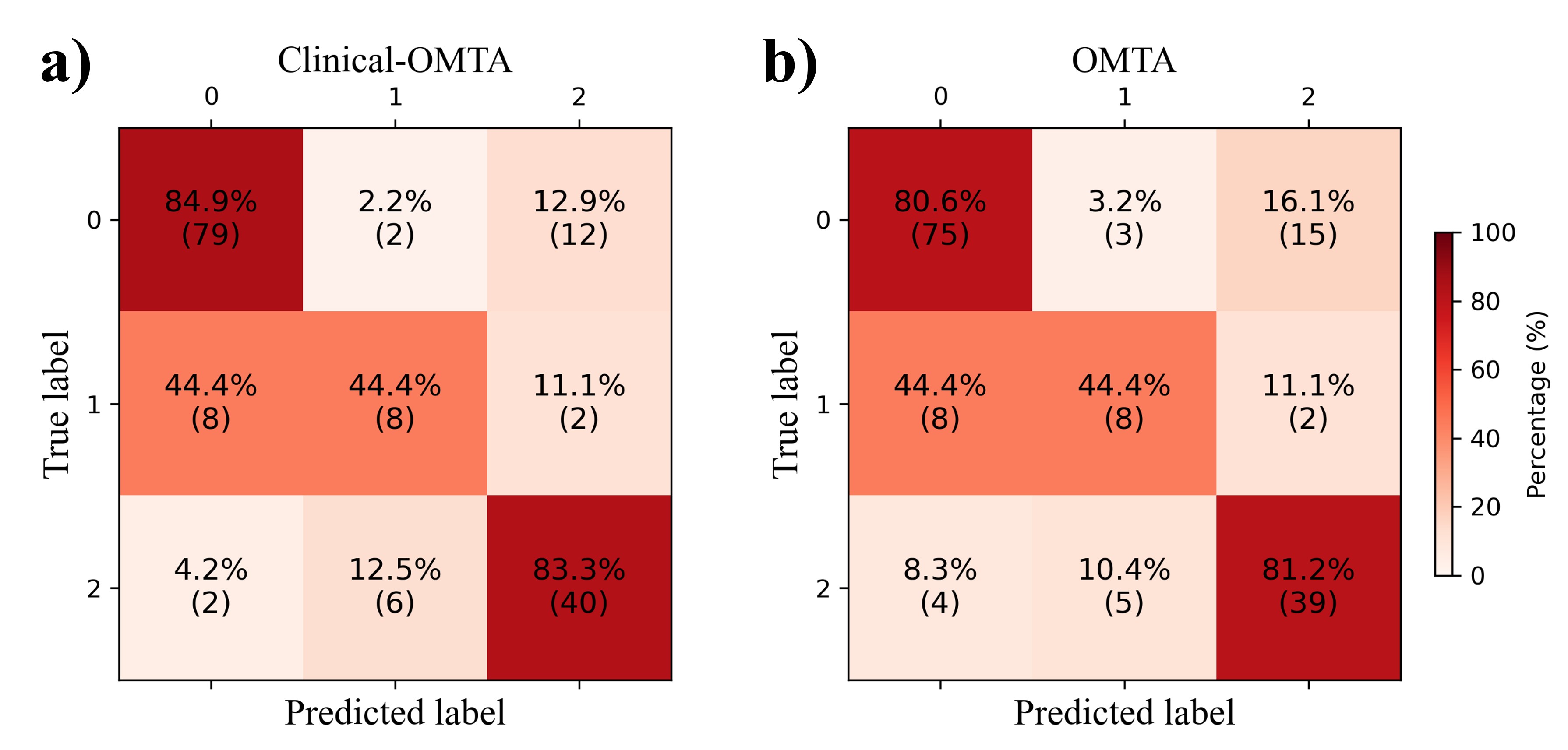


Figure S5. Confusion matrices of external video test dataset with expert manually correcting show the diagnostic performance of Clinical-OMTA (a) and OMTA (b) in discriminating benign, borderline and malignant adnexal tumours based on videos. Confusion matrices were based on 159 patient-level predictions. OMTA = Ovarian multi-task attention model, Clinical-OMTA = Clinical ovarian multi-task attention model.

**
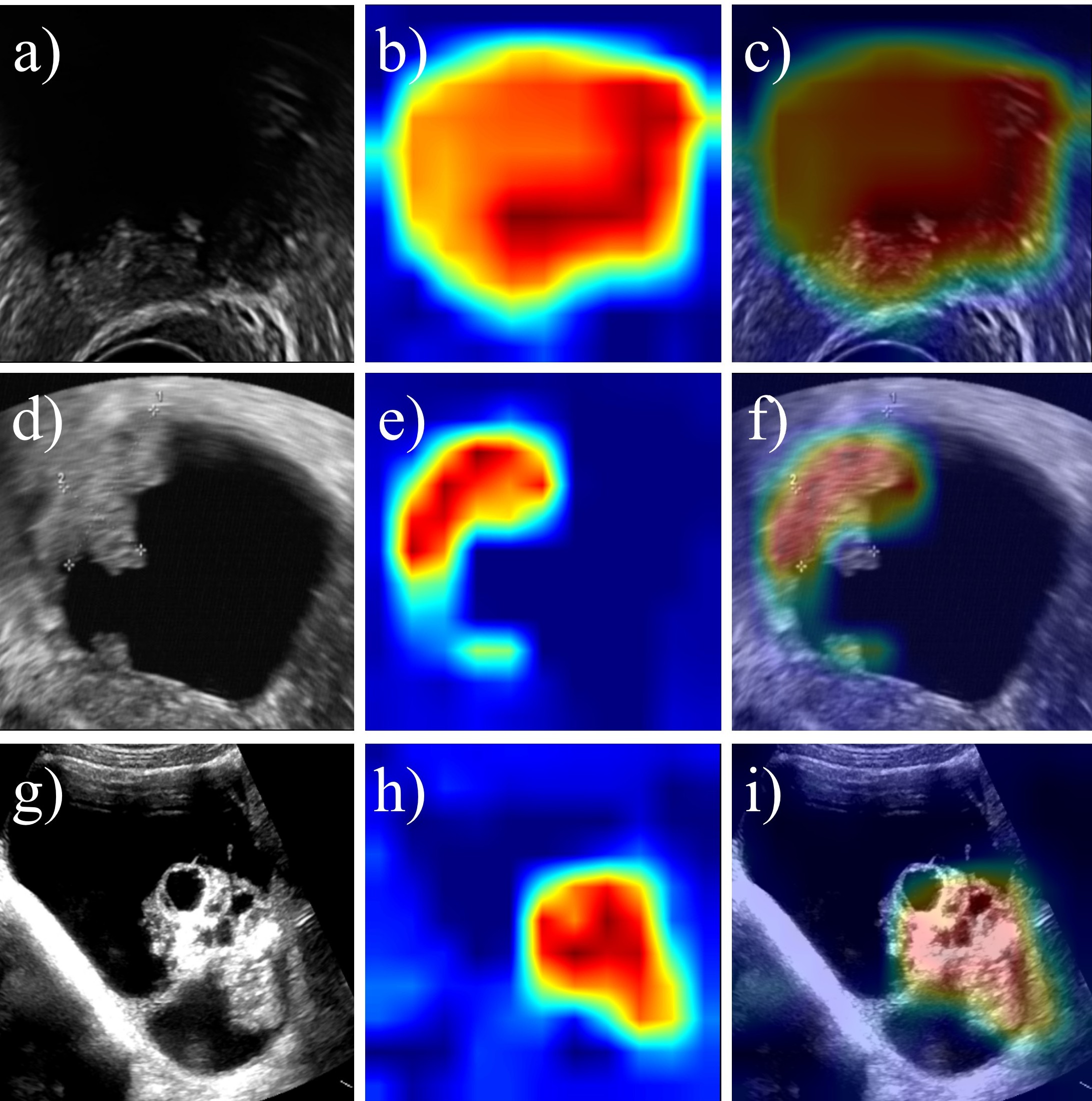
**

**Figure S6**. **Visualisation and interpretation of benign, borderline, and malignant adnexal masses.** **(a)** Ultrasound image in a 65-year-old female patient with no relevant symptoms (CA125 = 7.9 U/ml) shows a solid mass with acoustic shadow (benign Brenner tumour). **(b)** Heat map generated by OMTA shows the corresponding adnexal mass in (a). The classification model determines whether the mass is benign, borderline or malignant based on the image features from the highlighted region. **(c)** Overlapped ultrasound-heat map image shows OMTA focuses on shadowing of the solid mass. **(d)** Ultrasound image in a 46-year-old female patient with no relevant symptoms (CA125 = 62.2 U/ml) shows a cystic-solid mass (borderline serous cystadenoma). **(e)** Heat map generated by OMTA shows the corresponding adnexal mass in (d). **(f)** Overlapped ultrasound-heat map image shows that OMTA focuses on two papillary structures. **(g)** Ultrasound image in a 19-year-old female patient with no relevant symptoms (CA125 = 10.5 U/ml) shows cystic-solid mass (immature teratoma). **(h)** Heat map generated by OMTA shows the corresponding adnexal mass in (g). **(i)** Overlapped ultrasound-heat map image shows that OMTA focuses on solid component. Heat maps generated using the Gradient-weighted Class Activation Mapping. The heat map examples shown here were purposefully selected to demonstrate cases where the highlighted regions aligned well with expected anatomy. These panels are intended to be illustrative rather than representative of all generated heat maps. OMTA = Ovarian multi-task attention model, Clinical-OMTA = Clinical ovarian multi-task attention model. CA125 = Carbohydrate antigen 125.


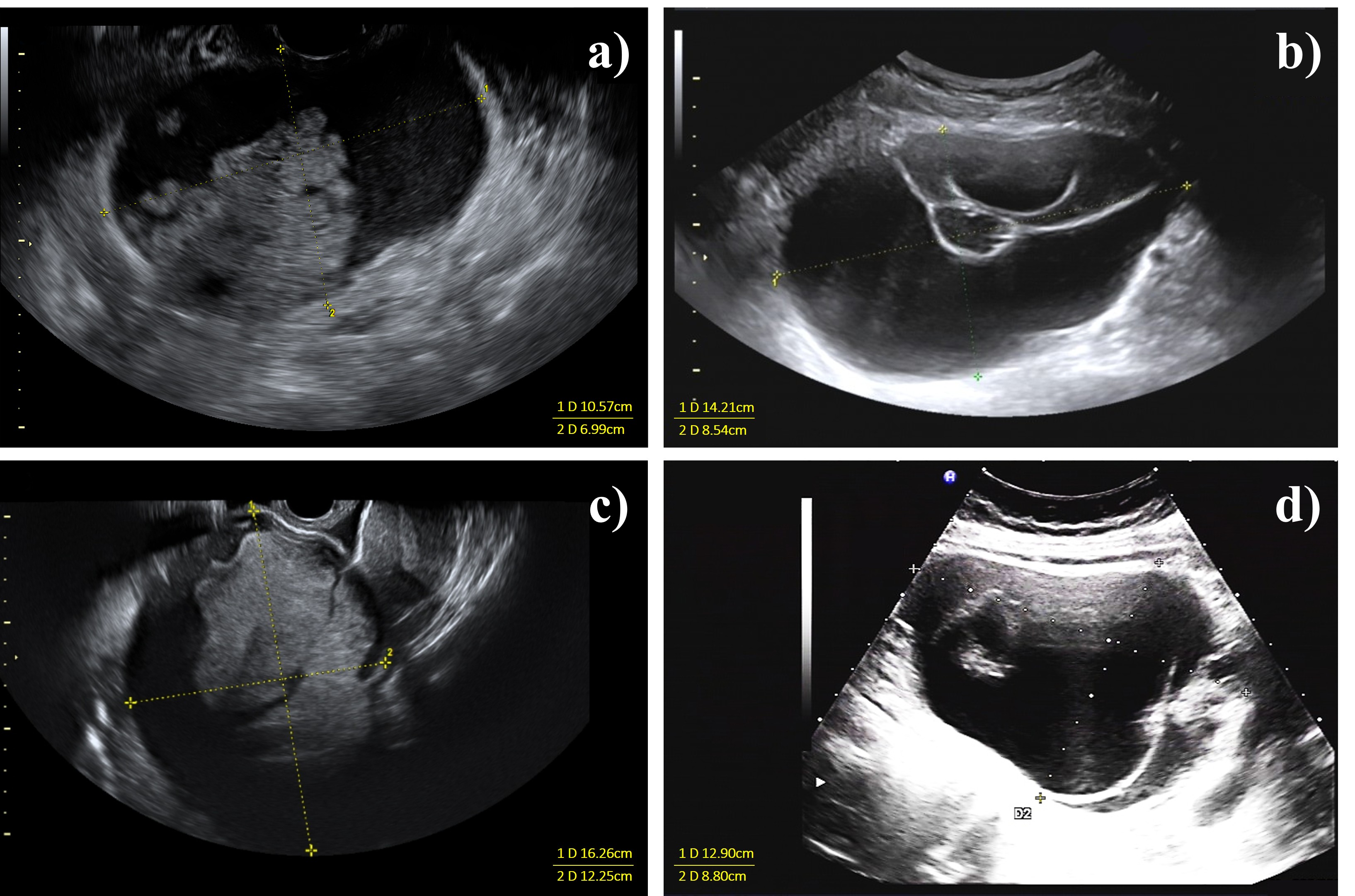


**Figure S7**. **Examples of ultrasound images of different types of adnexal masses. (a)** A 31-year-old female with a left ovarian solid-cystic mass without CA125 testing. The greyscale ultrasound image shows the mass at its largest size, with a pathological diagnosis of borderline mucinous cystadenoma. The Clinical-OMTA model and expert subjective assessment identified it as borderline, while the ADNEX model and eight out of ten less experienced radiologists classified it as malignant. After AI-assisted diagnosis, six out of ten less experienced radiologists made correct diagnoses; **(b)** A 30-year-old female with a left ovarian cystic mass, CA125 = 14.7 U/mL. The greyscale ultrasound image shows the mass at its largest size, with a pathological diagnosis of borderline mucinous tumour. The Clinical-OMTA model provided a correct diagnosis, whereas the ADNEX model, expert, and seven out of ten less experienced radiologists diagnosed it as benign. Following AI-assisted diagnosis, eight out of ten radiologists correctly identified it as borderline; **(c)** A 38-year-old female with a left ovarian solid-cystic mass, CA125 = 34.0 U/mL. The greyscale ultrasound image shows the mass at its largest size, with a pathological diagnosis of serous cystadenoma. The Clinical-OMTA model provided a correct diagnosis, while the ADNEX model and expert diagnosed it as malignant, and seven out of ten less experienced radiologists classified it as malignant or borderline. After AI-assisted diagnosis, nine out of ten radiologists correctly identified it as benign; **(d)** A 62-year-old female with a left ovarian solid-cystic mass, CA125 = 23.7 U/mL. The greyscale ultrasound image shows the mass at its largest size, with a pathological diagnosis of mucinous cystadenocarcinoma. The Clinical-OMTA model provided a correct diagnosis, whereas the ADNEX model, expert, and eight out of ten less experienced radiologists diagnosed it as benign. Following AI-assisted diagnosis, nine out of ten radiologists made correct diagnoses. Clinical-OMTA = Clinical ovarian multi-task attention model, ADNEX = Assessment of Different NEoplasias in the adneXa, CA125 = Carbohydrate antigen 125.

**
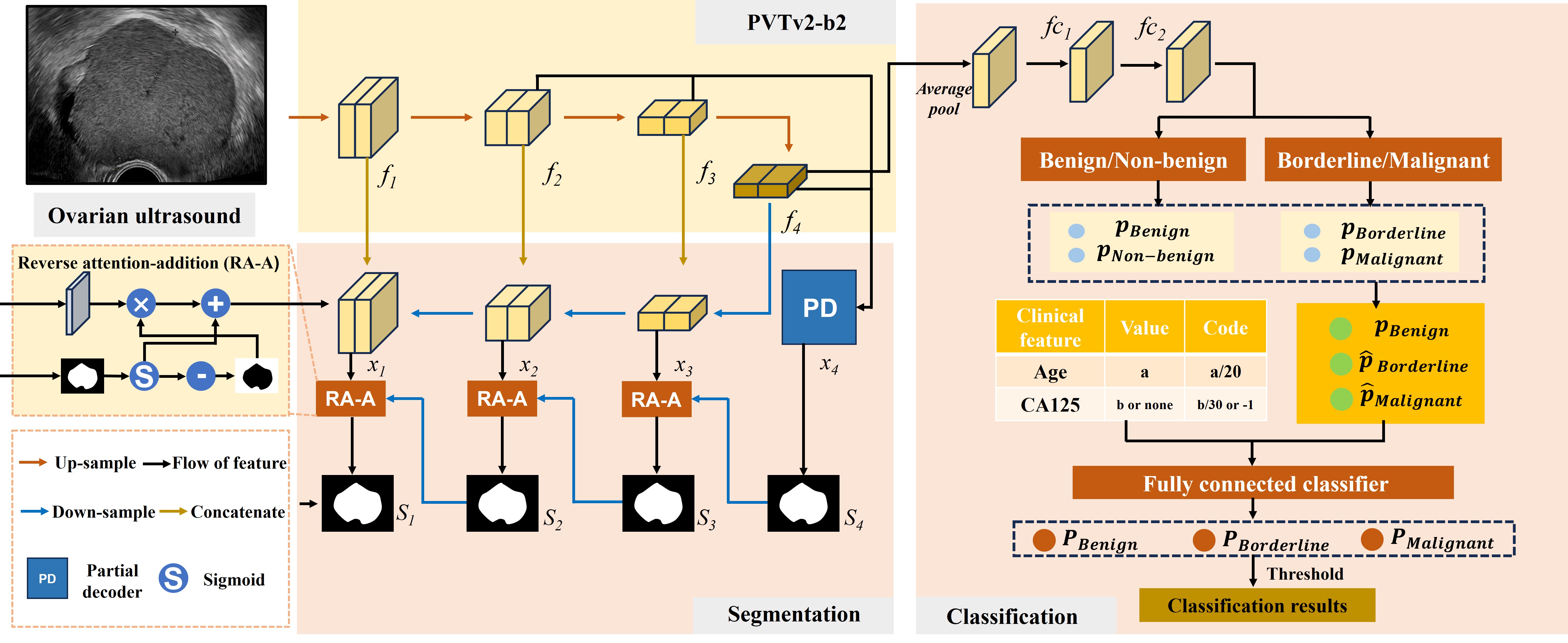
**

Figure S8. The proposed Clinical-OMTA outline. The network consists of a backbone encoder, a segmentation branch, and a classification branch. The backbone encoder extracts low-resolution features (*f_1_*, *f_2_*, *f_3_*, *f_4_*) from the input image. The segmentation branch uses a RA-A module to fuse area and boundary cues, generating high-resolution segmentation masks (*S_1_*, *S_2_*, *S_3_*, *S_4_*). The classification branch uses an attention bottleneck module to fuse image features and clinical data (age and CA125) for final classification. The “Value” column represents the original clinical data, while the “Code” column shows the standardised values used for model input after preprocessing. CA125 = Carbohydrate antigen 125, Clinical-OMTA = Clinical ovarian multi-task attention model, PVTv2-b2 = Pyramid vision transformer v2-b2, RA-A = Reverse addition attention.


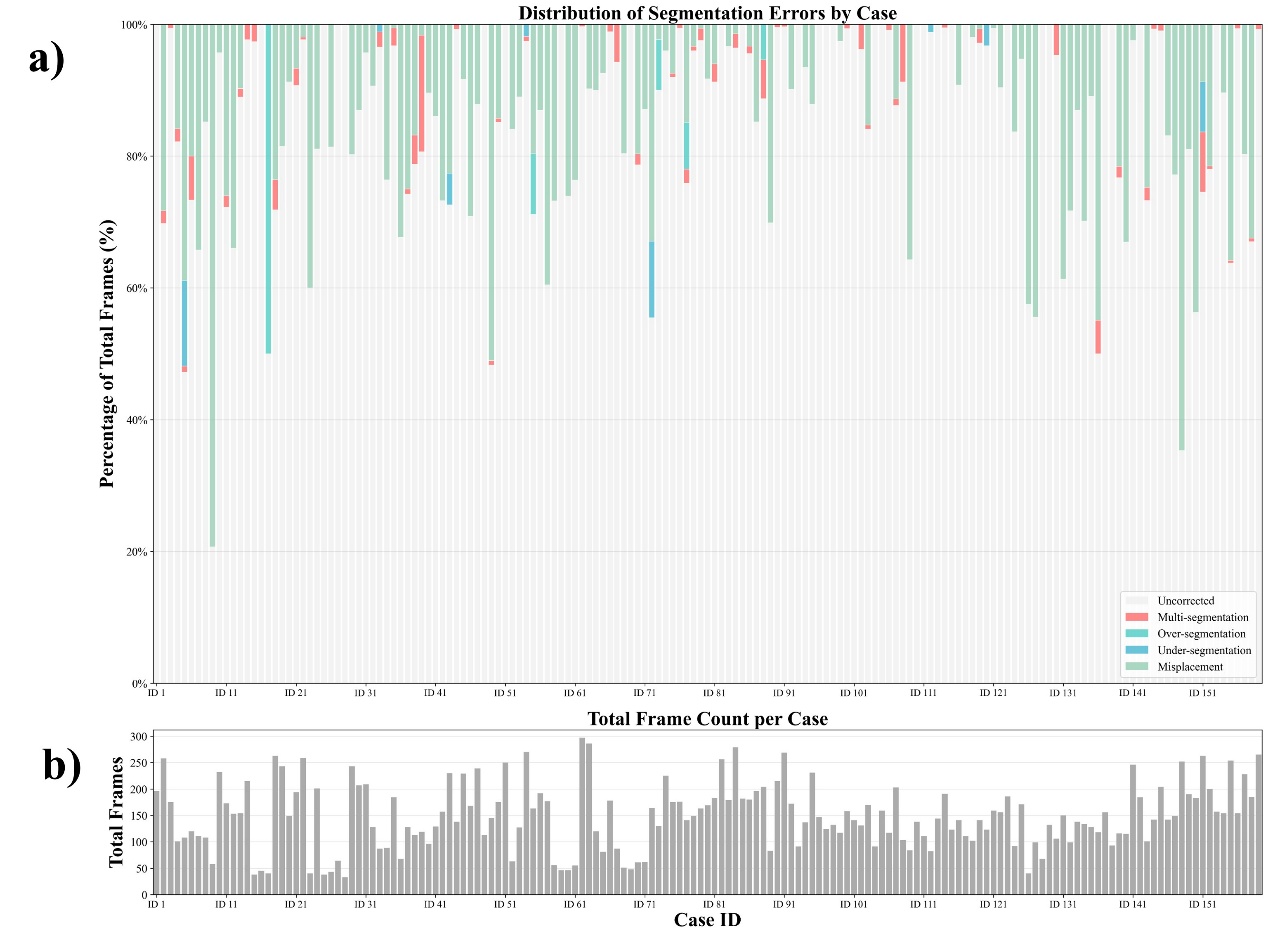


Figure S9. Visualisation of segmentation error distribution and total frame counts across 159 cases in the external video test dataset. (a) Stacked bar chart shows the proportional distribution of segmentation errors for each case. Each vertical bar represents one case with the total height normalised to 100%. The color-coded segments represent: Uncorrected Frames (Light Gray), frames requiring no correction; Multi-segmentation (Red), frames corrected for multiple segmentations of the same object; Over-segmentation (Cyan), frames corrected for oversized segmentation regions; Under-segmentation (Blue), frames corrected for undersized segmentation regions; Misplacement (Green), frames corrected for incorrectly placed segmentations. (b) Bar chart displays the absolute total frame count for each case, providing context for the relative scale of each dataset. The x-axis shows case ID numbers (1–159), aligned between both panels. This visualisation corresponds to the manual quality control step described in the methodology, which addressed potential segmentation errors, including under-segmentation, over-segmentation, misplacement, and multi-segmentation. The combined visualisation facilitates comparison of both proportional error distribution and absolute data volume across cases, offering comprehensive insights into the performance and limitations of the automated segmentation process. The frame rates of the included ultrasound videos ranged from 11 to 59 fps, reflecting the variation in acquisition settings across different clinical systems and examination protocols.

**STARD-2015-Checklist**

| Section & Topic | No | Item | Reported on page |
| --- | --- | --- | --- |
| TITLE OR ABSTRACT | | | |
|  | 1 | Identification as a study of diagnostic accuracy using at least one measure of accuracy (such as sensitivity, specificity, predictive values, or AUC) | 1 |
| ABSTRACT | | | |
|  | 2 | Structured summary of study design, methods, results, and conclusions (for specific guidance, see STARD for Abstracts) | 1 |
| INTRODUCTION | | | |
|  | 3 | Scientific and clinical background, including the intended use and clinical role of the index test | 2-3 |
|  | 4 | Study objectives and hypotheses | 3-4 |
| METHODS | | | |
| *Study design* | 5 | Whether data collection was planned before the index test and reference standard were performed (prospective study) or after (retrospective study) | 10 |
| *Participants* | 6 | Eligibility criteria | 10 |
|  | 7 | On what basis potentially eligible participants were identified (such as symptoms, results from previous tests, inclusion in registry) | 10 |
|  | 8 | Where and when potentially eligible participants were identified (setting, location and dates) | 10 |
|  | 9 | Whether participants formed a consecutive, random or convenience series | 10 |
| *Test methods* | 10a | Index test, in sufficient detail to allow replication | 10-14 |
|  | 10b | Reference standard, in sufficient detail to allow replication | 10 |
|  | 11 | Rationale for choosing the reference standard (if alternatives exist) | 10 |
|  | 12a | Definition of and rationale for test positivity cut-offs or result categories of the index test, distinguishing pre-specified from exploratory | 12 |
|  | 12b | Definition of and rationale for test positivity cut-offs or result categories of the reference standard, distinguishing pre-specified from exploratory | 10 |
|  | 13a | Whether clinical information and reference standard results were available to the performers/readers of the index test | 13 |
|  | 13b | Whether clinical information and index test results were available to the assessors of the reference standard | 13 |
| *Analysis* | 14 | Methods for estimating or comparing measures of diagnostic accuracy | 14 |
|  | 15 | How indeterminate index test or reference standard results were handled | (N/A) |
|  | 16 | How missing data on the index test and reference standard were handled | 11 |
|  | 17 | Any analyses of variability in diagnostic accuracy, distinguishing pre-specified from exploratory | 14 |
|  | 18 | Intended sample size and how it was determined | (N/A) |
| RESULTS | | | |
| *Participants* | 19 | Flow of participants, using a diagram | 4 |
|  | 20 | Baseline demographic and clinical characteristics of participants | 4 |
|  | 21a | Distribution of severity of disease in those with the target condition | (N/A) |
|  | 21b | Distribution of alternative diagnoses in those without the target condition | (N/A) |
|  | 22 | Time interval and any clinical interventions between index test and reference standard | 10 |
| *Test results* | 23 | Cross tabulation of the index test results (or their distribution) by the results of the reference standard | 22-23 |
|  | 24 | Estimates of diagnostic accuracy and their precision (such as 95% confidence intervals) | 4-6 |
|  | 25 | Any adverse events from performing the index test or the reference standard | (N/A) |
| DISCUSSION | | | |
|  | 26 | Study limitations, including sources of potential bias, statistical uncertainty, and generalisability | 9 |
|  | 27 | Implications for practice, including the intended use and clinical role of the index test | 6-9 |
| OTHER INFORMATION | | | |
|  | 28 | Registration number and name of registry | (N/A) |
|  | 29 | Where the full study protocol can be accessed | 15 |
|  | 30 | Sources of funding and other support; role of funders | 15 |

**TRIPOD+AI-checklist**

| **Section/Tozpic Item** | | **Development/ evaluation**1 | **Checklist item** | **Reported on page** |
| --- | --- | --- | --- | --- |
| TITLE | | | | |
| *Title* | 1 | D;E | Identify the study as developing or evaluating the performance of a multivariable prediction model, the target population, and the outcome to be predicted | 1 |
| **ABSTRACT** | | | | |
| *Abstract* | 2 | D;E | See TRIPOD+AI for Abstracts checklist | 1 |
| **INTRODUCTION** | | | | |
| *Background* | 3a | D;E | Explain the healthcare context (including whether diagnostic or prognostic) and rationale for developing or evaluating the prediction model, including references to existing models | 2-3 |
|  | 3b | D;E | Describe the target population and the intended purpose of the prediction model in the context of the care pathway, including its intended users (e.g., healthcare professionals, patients, public) | 2-3 |
|  | 3c | D;E | Describe any known health inequalities between sociodemographic groups | 3 |
| *Objectives* | 4 | D;E | Specify the study objectives, including whether the study describes the development or validation of a prediction model (or both) | 3-4 |
| **METHODS** | | | | |
| *Data* | 5a | D;E | Describe the sources of data separately for the development and evaluation datasets (e.g., randomised trial, cohort, routine care or registry data), the rationale for using these data, and representativeness of the data | 10 |
|  | 5b | D;E | Specify the dates of the collected participant data, including start and end of participant accrual; and, if applicable, end of follow-up | 10 |
| *Participants* | 6a | D;E | Specify key elements of the study setting (e.g., primary care, secondary care, general population) including the number and location of centres | 10 |
|  | 6b | D;E | Describe the eligibility criteria for study participants | 10 |
|  | 6c | D;E | Give details of any treatments received, and how they were handled during model development or evaluation, if relevant | (N/A) |
| *Data preparation* | 7 | D;E | Describe any data pre-processing and quality checking, including whether this was similar across relevant sociodemographic groups | 11 |
| *Outcome* | 8a | D;E | Clearly define the outcome that is being predicted and the time horizon, including how and when assessed, the rationale for choosing this outcome, and whether the method of outcome assessment is consistent across sociodemographic groups | 12-13 |
|  | 8b | D;E | If outcome assessment requires subjective interpretation, describe the qualifications and demographic characteristics of the outcome assessors | 13-14 |
|  | 8c | D;E | Report any actions to blind assessment of the outcome to be predicted | 13 |
| *Predictors* | 9a | D | Describe the choice of initial predictors (e.g., literature, previous models, all available predictors) and any pre-selection of predictors before model building | 14 |
|  | 9b | D;E | Clearly define all predictors, including how and when they were measured (and any actions to blind assessment of predictors for the outcome and other predictors) | 13 |
|  | 9c | D;E | If predictor measurement requires subjective interpretation, describe the qualifications and demographic characteristics of the predictor assessors | 13 |
| *Sample size* | 10 | D;E | Explain how the study size was arrived at (separately for development and evaluation), and justify that the study size was sufficient to answer the research question. Include details of any sample size  calculation | 10 |
| *Missing data* | 11 | D;E | Describe how missing data were handled. Provide reasons for omitting any data | 11 |
| *Analytical methods* | 12a | D | Describe how the data were used (e.g., for development and evaluation of model performance) in the analysis, including whether the data were partitioned, considering any sample size requirements | 11 |
|  | 12b | D | Depending on the type of model, describe how predictors were handled in the analyses (functional form, rescaling, transformation, or any standardisation). | 11 |
|  | 12c | D | Specify the type of model, rationale2, all model-building steps, including any hyperparameter tuning, and method for internal validation | 11-12 |
|  | 12d | D;E | Describe if and how any heterogeneity in estimates of model parameter values and model performance was handled and quantified across clusters (e.g., hospitals, countries). See TRIPOD-Cluster for additional considerations^3^ | 13 |
|  | 12e | D;E | Specify all measures and plots used (and their rationale) to evaluate model performance (e.g., discrimination, calibration, clinical utility) and, if relevant, to compare multiple models | 12-14 |
|  | 12f | E | Describe any model updating (e.g., recalibration) arising from the model evaluation, either overall or for particular sociodemographic groups or settings | (N/A) |
|  | 12g | E | For model evaluation, describe how the model predictions were calculated (e.g., formula, code, object, application programming interface) | 12-14 |
| *Class imbalance* | 13 | D;E | If class imbalance methods were used, state why and how this was done, and any subsequent methods to recalibrate the model or the model predictions | (N/A) |
| *Fairness* | 14 | D;E | Describe any approaches that were used to address model fairness and their rationale | (N/A) |
| *Model output* | 15 | D | Specify the output of the prediction model (e.g., probabilities, classification). Provide details and rationale for any classification and how the thresholds were identified | 12-14 |
| *Training versus evaluation* | 16 | D;E | Identify any differences between the development and evaluation data in healthcare setting, eligibility criteria, outcome, and predictors | 12-14 |
| *Ethical approval* | 17 | D;E | Name the institutional research board or ethics committee that approved the study and describe the participant-informed consent or the ethics committee waiver of informed consent | 10 |
| **OPEN SCIENCE** | | | | |
| *Funding* | 18a | D;E | Give the source of funding and the role of the funders for the present study | 15 |
| *Conflicts of interest* | 18b | D;E | Declare any conflicts of interest and financial disclosures for all authors | 15 |
| *Protocol* | 18c | D;E | Indicate where the study protocol can be accessed or state that a protocol was not prepared | 15 |
| *Registration* | 18d | D;E | Provide registration information for the study, including register name and registration number, or state that the study was not registered | (N/A) |
| *Data sharing* | 18e | D;E | Provide details of the availability of the study data | 15 |
| *Code sharing* | 18f | D;E | Provide details of the availability of the analytical code4 | 15 |
| **PATIENT & PUBLIC INVOLVEMENT** | | | | |
| *Patient & Public Involvement* | 19 | D;E | Provide details of any patient and public involvement during the design, conduct, reporting, interpretation, or dissemination of the study or state no involvement. | 15 |
| **RESULTS** | | | | |
| *Participants* | 20a | D;E | Describe the flow of participants through the study, including the number of participants with and without the outcome and, if applicable, a summary of the follow-up time. A diagram may be helpful. | 4 |
|  | 20b | D;E | Report the characteristics overall and, where applicable, for each data source or setting, including the key dates, key predictors (including demographics), treatments received, sample size, number of  outcome events, follow-up time, and amount of missing data. A table may be helpful. Report any differences across key demographic groups. | 4 |
|  | 20c | E | For model evaluation, show a comparison with the development data of the distribution of important predictors (demographics, predictors, and outcome). | 4 |
| *Model development* | 21 | D;E | Specify the number of participants and outcome events in each analysis (e.g., for model development, hyperparameter tuning, model evaluation) | 4 |
| *Model* *specification* | 22 | D | Provide details of the full prediction model (e.g., formula, code, object, application programming interface) to allow predictions in new individuals and to enable third-party evaluation and implementation, including any restrictions to access or re-use (e.g., freely available, proprietary)5 | 12-14 |
| *Model* *performance* | 23a | D;E | Report model performance estimates with confidence intervals, including for any key subgroups (e.g., sociodemographic). Consider plots to aid presentation. | 4-6 |
|  | 23b | D;E | If examined, report results of any heterogeneity in model performance across clusters. See TRIPOD Cluster for additional details3. | 5-6 |
| *Model updating* | 24 | E | Report the results from any model updating, including the updated model and subsequent performance | (N/A) |
| **DISCUSSION** | | | | |
| *Interpretation* | 25 | D;E | Give an overall interpretation of the main results, including issues of fairness in the context of the objectives and previous studies | 6 |
| *Limitations* | 26 | D;E | Discuss any limitations of the study (such as a non-representative sample, sample size, overfitting, missing data) and their effects on any biases, statistical uncertainty, and generalisability | 9 |
| *Usability of the* *model in the* *context of current care* | 27a | D | Describe how poor quality or unavailable input data (e.g., predictor values) should be assessed and handled when implementing the prediction model | 7 |
|  | 27b | D | Specify whether users will be required to interact in the handling of the input data or use of the model, and what level of expertise is required of users | 7-8 |
|  | 27c | D;E | Discuss any next steps for future research, with a specific view to applicability and generalisability of the model | 8-9 |

^1^ D=items relevant only to the development of a prediction model; E=items relating solely to the evaluation of a prediction model; D;E=items applicable to both the development and evaluation of a prediction model

^2^ Separately for all model building approaches.

^3^ TRIPOD-Cluster is a checklist of reporting recommendations for studies developing or validating models that explicitly account for clustering or explore heterogeneity in model performance (eg, at different hospitals or centres). Debray et al, BMJ 2023; 380: e071018 [DOI: 10.1136/bmj-2022-071018]

^4^ This relates to the analysis code, for example, any data cleaning, feature engineering, model building, evaluation.

^5^ This relates to the code to implement the model to get estimates of risk for a new individual.
